# Computationally Designed AMPs with Antibacterial and Antibiofilm Activity against MDR *Acinetobacter baumannii*

**DOI:** 10.3390/antibiotics12091396

**Published:** 2023-09-01

**Authors:** Fahad M. Alsaab, Scott N. Dean, Shravani Bobde, Gabriel G. Ascoli, Monique L. van Hoek

**Affiliations:** 1 School of Systems Biology, George Mason University, Manassas, VA 20110, USAshravani.bobde@gmail.com (S.B.); 2 College of Applied Medical Sciences, King Saud bin Abdulaziz University for Health Sciences, Al Ahsa 36428, Saudi Arabia; 3 Center for Bio/Molecular Science and Engineering, U.S. Naval Research Laboratory, Washington, DC 20375, USA; 4 Aspiring Scientist Summer Internship Program, George Mason University, Manassas, VA 20110, USA; 5 Center for Infectious Disease Research, George Mason University, Manassas, VA 20110, USA

**Keywords:** *Acinetobacter baumannii*, antimicrobial, peptide, multidrug-resistant bacteria, computational design, antibiofilm

## Abstract

The discovery of new antimicrobials is necessary to combat multidrug-resistant (MDR) bacteria, especially those that infect wounds and form prodigious biofilms, such as *Acinetobacter baumannii*. Antimicrobial peptides (AMPs) are a promising class of new therapeutics against drug-resistant bacteria, including gram-negatives. Here, we utilized a computational AMP design strategy combining database filtering technology plus positional analysis to design a series of novel peptides, named HRZN, designed to be active against *A. baumannii*. All of the HRZN peptides we synthesized exhibited antimicrobial activity against three MDR *A. baumannii* strains with HRZN-15 being the most active (MIC 4 µg/mL). This peptide also inhibited and eradicated biofilm of *A. baumannii* strain AB5075 at 8 and 16 µg/mL, which is highly effective. HRZN-15 permeabilized and depolarized the membrane of AB5075 rapidly, as demonstrated by the killing kinetics. HRZN 13 and 14 peptides had little to no hemolysis activity against human red blood cells, whereas HRZN-15, -16, and -17 peptides demonstrated more significant hemolytic activity. HRZN-15 also demonstrated toxicity to waxworms. Further modification of HRZN-15 could result in a new peptide with an improved toxicity profile. Overall, we successfully designed a set of new AMPs that demonstrated activity against MDR *A. baumannii* using a computational approach.

## 1. Introduction

The World Health Organization, the CDC, and the Pew Charitable Trust all describe the current lack of new antibiotics and emerging antibiotic resistance as a crisis. There is an urgent need for new antibiotics that are active against multidrug-resistant (MDR) bacteria and biofilm-forming wound infections. Antimicrobial peptides (AMPs) are a promising source for the development of innovative and potent antibiotics and antibiofilm compounds. The World Health Organization has specified the pathogens that are a critical and high priority for the development of novel antimicrobial agents [1,2]. These bacteria are commonly associated with biofilm-mediated wound infections such as combat wounds, blast wounds, burns, and chronic non-healing ulcers [3,4]. This group (called “ESKAPE” pathogens, from the first letter of each bacterium) includes both gram-positive and gram-negative bacteria and *Enterococcus faecium*, *Staphylococcus aureus*, *Klebsiella pneumoniae*, *Acinetobacter baumannii*, *Pseudomonas aeruginosa*, and *Enterobacter* species. These bacteria can infect wounds alone or in combination with other bacteria and are often multidrug-resistant. Biofilm is an extracellular sticky substance produced by bacteria that binds them to tissues and medical hardware, enables them to avoid antibiotics and the immune system, and directly aggravates the wound bed, prolonging and worsening the infection [5]. These bacteria alone and in mixed infections produce considerable amounts of biofilm as part of their pathology [6,7]. Often, these pathogens are antibiotic-resistant or multidrug-resistant (MDR) bacteria, which further complicates the treatment of these infections. Of special concern in the context of complex combat wounds is the emergence of MDR *A. baumannii* [3,8,9]. Murray et al. identified this organism in the infected wounds of many military personnel who participated in Operation Iraqi Freedom and other military operations [4,10,11,12]. *A. baumannii* is often highly multidrug-resistant, and thus is a specific target of our work [13], as new antibacterial approaches are critically needed.

Antimicrobial peptides (AMPs) are small peptide molecules (usually less than 50 amino acids) that can kill bacteria directly and/or inhibit the ability of bacteria to form biofilms. We have studied as well as invented multiple AMPs with direct antibacterial activity against these pathogens, including *S. aureus* [14,15,16], *K. pneumonia* [17], *A. baumannii* [16,17], and *P. aeruginosa* [14,16,18]. Our group has also identified AMPs with activity against biothreat pathogens *Francisella tularensis* [19,20,21], *Burkholderia thailandensis* [22], and *Bacillus anthracis* [23]. We have characterized many AMPs against a range of gram-positive and gram-negative human pathogens, including *A. baumannii* [6,14,15,16,17,18,19,20,22,23,24,25,26,27,28,29,30]. We have also identified and studied the activity of many AMPs with antibiofilm properties [14,18,19]. The human cathelicidin LL-37 has been extensively studied for host- and pathogen-directed functions. It has been previously demonstrated that LL-37 possesses antimicrobial activity against MDR *P. aeruginosa*, methicillin-resistant *S. aureus*, and vancomycin-resistant *E. faecium* [31]. The peptide was also shown to have antibacterial and antibiofilm activities against MDR *A. baumannii* [32]. The antibacterial activity of WLBU2 (PLG0206), a synthetic peptide that is rich in arginine, was tested, and results showed strong activity against extensively drug-resistant (XDR) *A. baumannii* and *K. pneumoniae* with low reported MIC [33]. This peptide is currently in Phase 1 clinical trial [34]. A synthetic and non-toxic analog of Pseudin-2, Pse-T2, was shown to be effective against MDR strains of *Escherichia coli*, *P. aeruginosa*, and *S. aureus* with biofilm-inhibitory effects [35]. The in vivo activity of Pse-T2 was assessed using a mouse wound model infected with MDR *P. aeruginosa*, and results showed improved healing of the wound accompanied by the eradication of the bacteria, as well as enhanced wound closure [35]. Pexiganan (MSI-78), an analog of magainin, is currently in Phase 3 clinical trial for treating diabetic foot infection [36]. The in vitro antibacterial testing of pexiganan against a panel of pathogens that cause diabetic foot infections showed the effectiveness of the peptide [36]. Previous work has been based on either discovered peptides or rational design (WLBU2). Our work seeks to expand this prior work by implementing computational peptide design.

There are a large number of natural AMPs that have been discovered in living organisms as well as synthetic AMPs that have been developed by rational design [37]. However, the implementation of computational approaches to generate antimicrobial peptides based on databases could result in the invention of many more novel compounds with improved antimicrobial activity [38]. Designing and testing computationally designed AMPs will expand the number of experimentally validated AMPs that could serve as templates for peptide design and be a part of high-quality datasets to aid further computational peptide design pipelines [39]. In previous work, we designed novel peptides (PHNX) using a combined computational strategy of database filtering technology (DFT) plus a positional analysis on a dataset derived from APD3 [37], and we were able to design a novel AMP with specific activity against gram-negative bacteria, called PHNX-8 [40]. 

In this work, we sought to computationally design new AMPs that would have activity against MDR *A. baumannii* using a similar computational approach. This series of peptides was named “HRZN” as in “horizon”, as they represent a new series of peptides that could potentially treat severe wound infections. Our hypothesis was that computationally designing a new peptide from a large set of peptides with reported activity against any strain of *A. baumannii* should increase the likelihood of obtaining peptides with activity against MDR *A. baumannii*, which is demonstrably 82% more challenging for an organism with all the efflux pumps and multiple drug-resistance mechanisms [41,42]. We assembled a curated set of peptides from the Giant Repository of AMP Activities (GRAMPA) database that have reported activity against any strain of *A. baumannii* [43]. The GRAMPA database contains data collected from various other databases, including APD [37], DADP [44], DBAASP [45], DRAMP [46], and YADAMP [47]. Our set of collected peptides (374) was analyzed using DFT plus positional analysis (“DFT plus PA”) to generate novel peptides that we predicted would be active AMPs. We then selected and tested the newly designed AMPs against multidrug-resistant (MDR) strains of *A. baumannii* for antibacterial, antibiofilm, and resistance acquisition, as well as evaluated them for hemolysis and toxicity.

## 2. Results

### 2.1. Design of Peptides

HRZN 13–17 was designed by database filtering technology (DFT) with the additional positional analysis (PA) filter to design a set of new peptides. The Giant Repository of AMP Activities (GRAMPA) database [43] consists of 6760 unique sequences and 51,345 MIC values against various organisms. We collected 643 peptides that reported activity against *A. baumannii* with reported MIC values. Dataset 1 (374 sequences) was obtained by removing duplicate sequences from that list. DFT was performed on Dataset 1 by MIC selection, and then size selection and positional analysis [40,48], which resulted in HRZN-13 and 14. Further processing using positional analysis based on different iterations yielded three additional peptides. HRZN-15, 16, and 17 were generated by processing on peptide lengths of 13, 13 ± 3, and 13 ± 4 amino acids, respectively [40]. All peptides were synthesized by ChinaPeptides with high purity (≥98%) and checked by reverse-phase high-performance liquid chromatography.

Secondary structures of the newly designed HRZN peptides (Table 1) were predicted by AlphaFold2, and all peptides were predicted to adopt α-helical structures [49]. The α-helical structure is linked to the ability of peptides to lyse membranes and contributes to antibacterial activity, as in magainin 2 [50], and was one of our design parameters. Analysis of physicochemical characteristics revealed that HRZN peptides have an overall hydrophobicity ratio of 54–62% of the amino acids and an overall positive charge of +3–+6. This is consistent with our previous results, which found that gram-negative active AMPs have hydrophobicity of around 50% and an optimal net charge between +2 and +4 [40]. HRZN-13 and -14 sequences differ in one residue at position 11 (K in HRZN-13 and R in HRZN-14); hence, they both have +5 charge and 54% total hydrophobicity. HRZN-15 and HRZN-16 have the highest hydrophobic ratio of 62% and the highest hydrophobic moment, but they differ in the net charge, with HRZN-15 having +3 and +5 for HRZN-16. HRZN-17 has the highest net charge of +6 and similar hydrophobicity to HRZN-13 and -14, but the highest hydrophobic moment. The Heliquest program (https://heliquest.ipmc.cnrs.fr, accessed on 29 September 2021) was used to generate the helical wheel projections, illustrating the amphipathicity and the hydrophobic face of each peptide and calculating the hydrophobic moment.

These newly designed peptides all represent new and novel sequences, none of which had been previously identified in any of the databases we searched. For example, the HRZN-15 sequence was searched in APD, DBAASP, and YADAMP, and there were no identical sequences. Peptide alignment of HRZN-15 was performed in APD and resulted in a 69.23% similarity of HRZN-15 to Temporin L and Temporin-1Ob.

### 2.2. Antimicrobial Activity Prediction

Prior to experimentally testing the activity of the peptides, the antimicrobial activity of HRZN peptides was computationally predicted using tools such as support vector machine (SVM), random forest (RF), artificial neural network (ANN), and discriminate analysis (DA). Comparing the output from different tools increases confidence in the results of our antimicrobial activity predictions. Lee et al. developed an SVM classifier (also known as a “Ferguson classifier”) that predicts the antimicrobial activity of α-helical peptides and their ability to generate negative Gaussian curvature, which is important in membrane permeability [51]. CAMPR3 predicts antimicrobial activity through SVM, RF, ANN, and DA models that utilize a positive dataset containing experimentally active AMPs and a negative dataset that contains inactive AMPs [52,53]. The ClassAMP antimicrobial prediction tool utilizes CAMP-positive and -negative datasets [54,55]. However, the difference lies in the positive dataset that is classified into antibacterial, antigungal, or antiviral, and the prediction results are generated based on these classes. The DBAASP antimicrobial predictor classifies linear sequences to be AMPs and non-AMPs based on a machine learning algorithm that uses physicochemical properties, such as hydrophobicity, and charge density and the propensity of a peptide interact with the membranes [56]. A classifier based on the XGBoost-based regression model described in Dean et al. [57] was also used to evaluate the likelihood that HRZN peptides were AMPs, where each peptide sequence was input and AMP or non-AMP was predicted based on sequence-based features.

In Table 2, we list the predicted antimicrobial activity for all our designed peptides. HRZN-13 and -14 have a high prediction score of 0.99 or 1 probability in the Ferguson classifier. CAMPR3 [53] and CLASSAMP [54] were predicted to be AMP in CAMPR3 (ANN), DBAASP, and PepVAE3 classifers followed by HRZN-15 and -16, which scored at least 0.96 in all algorithms and were predicted to be AMP. HRZN-17 had the lowest prediction score in CAMPR3 RF (probability 0.6) and achieved ≥ 0.93 across the other predictors with the potential to be antimicrobial. DBAASP, CAMPR3, and PepVAE3 predicted LL-37 to be an AMP, and notably, the probability scores of CAMPR3 for LL-37 range from 0.75 to 0.77. LL-37 scored ≥ 0.95 in the Ferguson classifier and CLASSAMP. IDR-1018 was predicted to be non-AMP by DBAASP and scored very poorly in the Ferguson classifier (0.15). However, the CAMPR3 and CLASSAMP prediction results gave a probability of 0.97–0.99 for IDR-1018. These results suggest that our HRZN peptides will likely be antimicrobial, with the possible exception of HRZN-17. They also reveal some of the issues with these predictors, such as the disagreements between them for the same peptide, and disagreement with actual experimental results, such as for IDR-1018.

### 2.3. Antimicrobial Susceptibility Testing

The peptides in Table 1 were screened for antibacterial activity. The benefit of screening peptides for activity first before performing a full MIC analysis for each one is to identify those that possess inhibitory effects at a relatively high concentration (100 μg/mL) and eliminate or downselect any inactive peptides. HRZN peptides were screened for antimicrobial activity at 100 µg/mL against a panel of multidrug-resistant *A. baumannii* strains to quickly identify viable peptides for further analysis. All peptides inhibited the growth of MDR *A. baumannii* strains. Peptides that exhibited a growth-inhibitory effect at 100 μg/mL were then assessed in detail via an MIC assay against *A. baumannii* strains AB5075, BAA-1710, -1794, and -1800. 

After screening, a minimum inhibitory concentration assay, which measures the lowest concentration of a compound that inhibits the growth of bacteria, was performed on HRZN peptides against four *A. baumannii* strains. These strains were selected in this study because they were isolated from different infection sites in humans including bone, blood, sputum, and trachea. Also, the strains have different antibiotic resistance profiles (e.g., AB5075 is susceptible to tetracycline, while BAA-1710, -1794, and -1800 are not) [58,59,60,61]. AB5075 is proposed to be an MDR model strain for studying pathogenesis and antimicrobial testing [61].

We followed the standardized method for testing AMP activity [62]. The strain AB5075 is highly resistant to multiple antibiotics including Amp/Sul, Amk, Azt, Cfp, Cfz, Cpr, Gen, Imi, Lev, and Tob [61]. HRZN-15 had the strongest antimicrobial activity, resulting in an MIC of 4 µg/mL (Table 3), followed by HRZN-16 and HRZN-17, which demonstrated an MIC of 16 and 32 µg/mL (Table 3) across the tested strains, respectively. On the other hand, HRZN-13 showed the weakest activity MIC 32–64 µg/mL (Table 3), and HRZN-14 exhibited variable MIC ranging from 16 to 64 µg/mL (Table 3). This result was not predicted by the computational analysis, which suggested that HRZN-17 might have the poorest activity. LL-37 and polymyxin B were used as controls for MIC determination and showed bacterial growth inhibition at 8–32 and 0.25–0.5 µg/mL (Table 3), respectively, which was in agreement with reported results, except for the MIC value of LL-37 against AB5075 [32,63]. The full data for the MIC assays of *A. baumannii* strains AB5075, BAA-1710, -1794, and -1800 are in the Supplementary Materials (Figures S2–S5). 

### 2.4. Time-Kill Kinetics

Another way to assess the activity of the peptides is to measure the killing activity of peptides over time rather than over a range of peptide concentrations, as in MIC. The more rapidly a peptide kills bacteria, the more active it is likely to be. Since HRZN-15 showed strong antimicrobial activity, we measured its time-killing kinetics. HRZN-15 peptide was tested at 1× MIC, 5× MIC, and 10× MIC, considering the MIC to be 8 µg/mL for AB5075 and 4 µg/mL for BAA-1710. Bacterial killing was assessed for AB5075 and BAA-1710 over three hours. Polymyxin B was used as a control.

**Figure 1:**
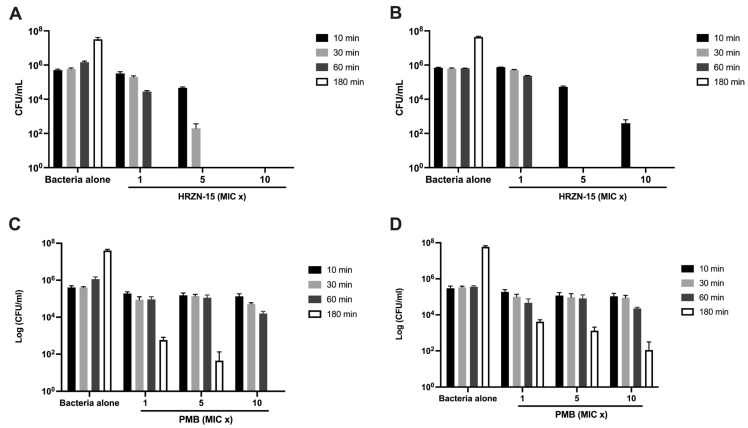
Time-kill kinetics of HRZN-15 and polymyxin B. Inoculum of 5 × 10^5^ CFU/mL of (**A**,**C**) AB5075 and (**B**,**D**) BAA-1710 were challenged with (**A**,**B**) HRZN-15 at MIC = 8 and 4 µg/mL and (**C**,**D**) polymyxin B at MIC = 0.5 µg/mL to determine their killing activity over 10, 30, 60, and 180 min. “MIC x” indicates the multiple of MIC used. Bars that are not visible indicate 0 CFU/mL.

The killing kinetics of HRZN-15 against *A. baumannii* (Figure 1A,B) revealed the peptide’s ability to completely kill at a 1× MIC concentration after 3 h, irrespective of the strain tested, which is very fast. At 5× MIC, HRZN-15 eliminated AB5075 after 1 h and BAA-1710 after 30 min. The peptide also showed rapid killing activity within 10 min at 10× MIC against AB5075. HRZN-15 was equally effective in time-dependent killing activity when 10× MIC was tested against BAA-1710 compared to 5× MIC, both at 30 min. The “drug of last resort”, polymyxin B, only killed AB5075 (Figure 1C) within 3 h at the highest concentration (5 µg/mL), but it failed to kill the BAA-1710 strain within 3 h at all tested concentrations (Figure 1D). These results demonstrate that HRZN-15 acts very quickly compared to polymyxin B. Recent reports have demonstrated similarly rapid killing kinetics of other peptides [64,65]. 

### 2.5. Antibiofilm Assays

The HRZN peptides were first screened for their biofilm inhibition activity. All the peptides demonstrated complete inhibition of *A. baumannii* BAA-1710 and -1794 biofilm at 100 µg/mL. LL-37 and polymyxin B were used as the controls [18,66,67] and showed similarly complete biofilm inhibition to HRZN peptides.

#### 2.5.1. Minimum Biofilm Inhibitory Concentration (MBIC)

Since HRZN-15 exhibited strong antibacterial activity and inhibited biofilm formation in the screening assay, it was selected for minimum biofilm inhibitory concentration (MBIC) experiment. *A. baumannii* strain AB5075 was tested for biofilm formation but did not produce sufficient biofilm mass for the MBIC assay [32]. Instead, *A. baumannii* BAA-1800 was used in these MBIC assays as it formed more robust biofilms. MBIC measures the lowest concentration of a compound that inhibits bacterial biofilm formation. HRZN-15 was able to inhibit biofilm of *A. baumannii* BAA-1800 at a slightly higher concentration (8 g/mL, Table 4) than MIC, which is 4 µg/mL (Figure S6A), (MBIC_50_ ~4 µg/mL) suggesting that the mechanism is likely bacteria-killing rather than directly inhibiting extracellular polymeric substance (EPS) production. Additional results are presented in Figure S7. LL-37 inhibited *A. baumannii* BAA-1800 biofilm production at 64 µg/mL (Figure S6B) (MBIC_50_ ~32 µg/mL), even though it did not inhibit bacterial growth at this concentration, supporting a more direct inhibition of biofilm formation (Figure S7B). This effect has been previously shown for LL-37 against other gram-negative bacteria, like *Pseudomonas aeruginosa* [18,67]. Peptide IDR-1018 showed a dose-dependent biofilm inhibition of *A. baumannii* BAA-1800 (Figure S7C) (MBIC_50_ ~32 µg/mL) that was also separate from bacterial growth inhibition (Figure S7C), illustrating that IDR-1018 is able to directly modulate biofilm production separately from its antibacterial activity, likely via its regulation of ppGpp production, as previously reported [66]. Our results for IDR 1018 (Table 4) differ somewhat from de la Fuentez, 2014 [66], who determined IDR-1018 MBIC_50_ = 2 µg/mL, MBIC_100_ = 10 µg/mL, and MIC = 128 µg/mL for *A. baumannii* SENTRY C8 strain, which may reflect strain-dependent differences or broth differences, as they were performed in Basal Medium 2 (BM2), unlike our study in which Tryptic Soy Broth was used. Polymyxin B demonstrated an MBIC of 1 µg/mL (Figure S6D), as previously shown against different strains through the biofilm prevention assay [68]. The antibiofilm concentration of HRZN peptides is very similar to the antibacterial concentration, illustrating that this peptide can kill MDR *A. baumannii*, even when it is in a biofilm. 

As a side note, it was observed that biofilm mass increases at low peptide concentrations compared to untreated wells, as seen in Figure S6 The mechanism of this effect is unknown but has been observed for other peptides and other bacteria [15,18,22,23]. 

#### 2.5.2. Minimum Biofilm Eradication Concentration (MBEC)

The HRZN-15 peptide, which demonstrated antibiofilm activity in the MBIC assay, was further tested for the eradication of pre-formed (24 h) biofilms through the MBEC assay. A minimum biofilm eradication concentration (MBEC) assay is defined as the lowest drug concentration that eradicates previously established biofilm. Using an MBEC assay plate (Innovotech), we were able to establish biofilms of AB5075 on the pegs for 24 h and stain them with crystal violet for biomass quantification, as previously shown [32]. HRZN-15 eradicated pre-formed biofilms of AB5075 with treatment at MBEC_100_ 16 µg/mL (Figure S8A, Table 4) with an MBEC_50_ of 8 µg/mL. LL-37 acted in a concentration-dependent manner against established biofilm, achieving 80% biofilm eradication at 32 µg/mL (Figure S8B) and MBEC_50_ at 4 µg/mL, which was consistent with published results [32]. At the highest IDR-1018 concentration tested (32 µg/mL), only 20% of biofilm was eradicated (Figure S8C), which was consistent with what was previously shown [66], demonstrating that this peptide can eradicate pre-formed biofilms in *A. baumannii*. 

Notably, biofilms treated with polymyxin B (Figure S8D) were persistent, maintaining up to 40% of crystal violet-stained biofilm mass when treated with between 2 and 16 µg/mL polymyxin B, although the MBIC_50_ was 2 µg/mL (Table 4). Due to this high level (40%) of biofilm staining in the crystal violet MBEC assay, we wanted to investigate if the stained biofilm mass contained bacteria or was just a residual stained extracellular matrix (ECM). A reduction in CFU was determined on polymyxin B-treated pre-formed biofilms, corresponding to Figure S8D. We demonstrated the complete eradication of bacteria at 2 µg/mL polymyxin B, illustrating a limitation of the crystal violet-based MBEC assay to detect residual ECM in the absence of viable bacteria.

### 2.6. Mechanism of Action, Scanning Electron Microscopy, and Resistance Induction

**Figure 2:**
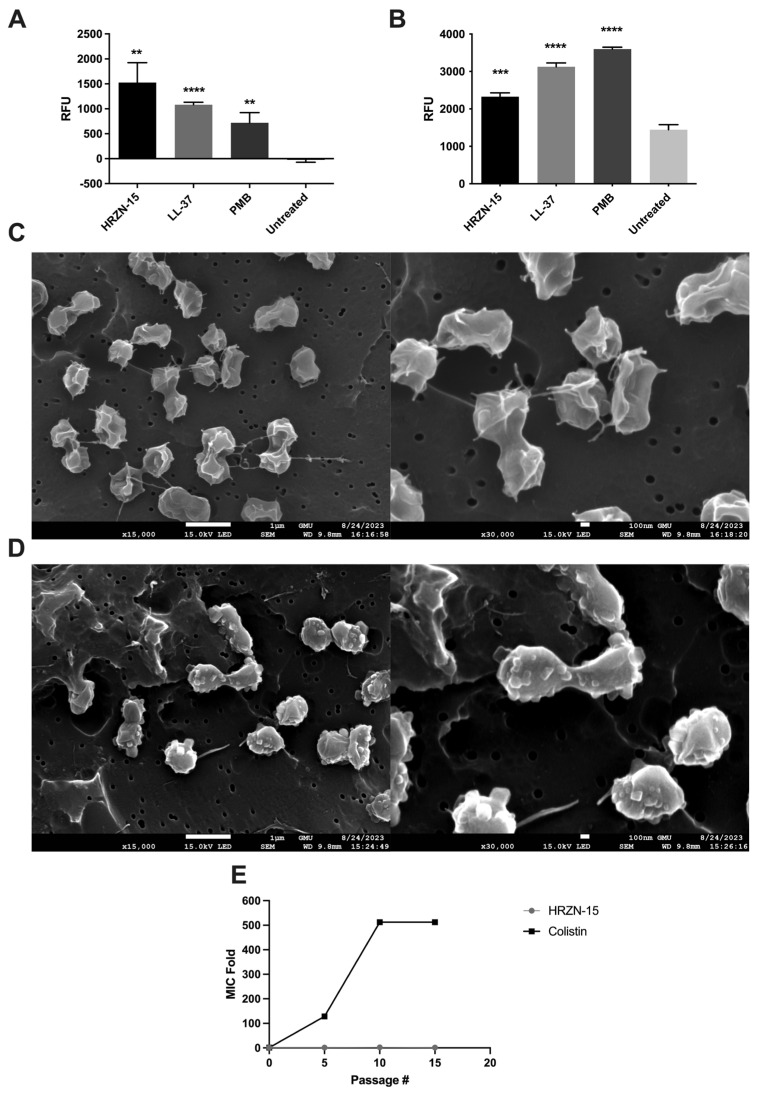
Mechanism of action, visualization of bacteria under SEM, and resistance induction of HRZN-15 against *A. baumannii* AB5075. (**A**) Membrane depolarization (DiSC_3_(5)) and (**B**) permeabilization (EtBr) of AB5075 upon exposure to 50 µg/mL of peptide or antibiotics after 20 min of incubation. SEM images of (**C**) untreated and (**D**) HRZN-15-treated AB5075. (**E**) In vitro resistance induction of AB5075 upon exposure to subinhibitory concentrations of HRZN-15 and colistin for 15 days. The figure shows an MIC fold change in HRZN-15 and colistin on peptide or antibiotic-exposed bacteria. “Passage #” indicates the passage number at which the MIC was determined. Statistical significance is indicated relative to the untreated samples by asterisks (** *p* < 0.01, *** *p* < 0.001, **** *p* < 0.0001).

The membrane depolarization assay, which utilizes the cationic fluorescent dye DiSC_3_(5) to evaluate the ability of peptides to damage bacterial membranes causing ion leakage, of HRZN-15 was assessed against AB5075 upon exposure to 50 µg/mL (Figure 2A). Although the MIC is 4 µg/mL, we tested for the mechanism of action at 50 µg/mL following our previous mechanistic studies [16,26,27,28,29]. HRZN-15 depolarized the bacterial membrane in a significant manner (*p* < 0.01) compared to the untreated bacteria. LL-37 and polymyxin B also showed a significant membrane depolarization effect. We previously demonstrated peptide depolarizing effect against bacteria [14,15,16,17,18,27]. The data for the kinetics of membrane depolarization are found in Figure S5. 

The loss of membrane integrity was assessed using ethidium bromide, a DNA intercalating agent, upon peptide exposure, as we have previously reported [14,15,16,17,18,26,27,28]. When large-scale disruptions in the membrane occur, the ethidium bromide can enter the bacterial cells and stain the DNA. AB5075 was challenged with 50 µg/mL of HRZN-15, LL-37, or polymyxin B, and fluorescence intensity was measured. Our results show that HRZN-15 permeabilized *A. baumannii* bacterial membrane (Figure 2B) as relative fluorescence units increased compared to an untreated control containing only bacteria and ethidium bromide (*p* < 0.001). The control antimicrobials LL-37 and polymyxin B also disrupted bacterial membranes, as we have shown previously [14,15,16,17,18,26,27,28]. The kinetics of membrane disruption revealed the rapid action of HRZN-15 on bacteria, as reflected at the first time point, time 0 (Figure S9), while LL-37 and polymyxin B affected the bacterial membrane much later. 

To understand the ability of the peptide to induce changes in the bacteria, a scanning electron microscope (SEM) was used to visualize the bacteria with or without peptide treatment. *A. baumannii* was treated with 50 µg/mL of HRZN-15 for 20 min. Untreated bacteria (Figure 2C) showed irregular morphology that might be attributable to shrinkage due to freeze drying (lyophilization). Conversely, HRZN-15-treated bacteria (Figure 2D) showed significant alterations in bacterial morphology compared to the control with course structures protruding from the bacteria, suggesting cell damage. 

One of the advantages of membrane lytic peptides is the difficulty for bacteria to acquire resistance due to the quick killing and fundamental nature of the membrane. We evaluated the induction of resistance of HRZN-15 and colistin upon exposure to subinhibitory levels. AB5075 acquired resistance rapidly after the fifth day of colistin exposure with increased MIC from 0.5 to 64 µg/mL (128 MIC fold) (Figure 2E). AB5075 resistance to colistin continued to increase, reaching 512× MIC compared to the initial growth-inhibitory concentration (Figure 2E), which was consistent with published results [50]. In contrast, HRZN-15 showed no induction of resistance, and MIC remained consistent throughout the exposure and passaging period, except at the 10th passage where the MIC increased to 8 µg/mL. The MIC results for each passage are shown in Figure S10.

### 2.7. Toxicity Assessment

**Figure 3:**
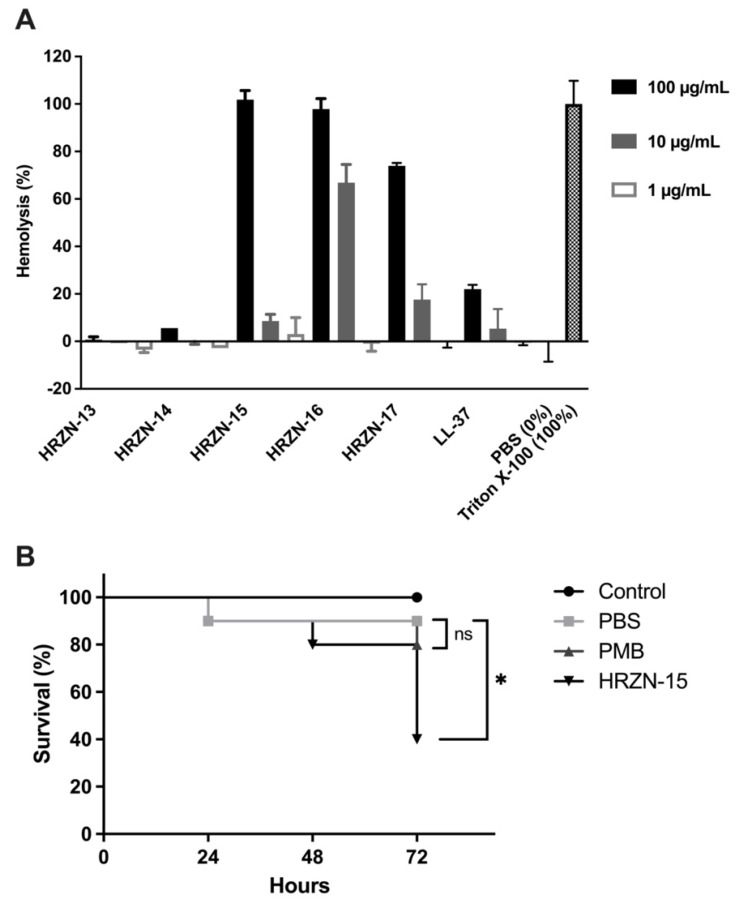
Toxicity assessment of HRZN peptides. (**A**) Hemolysis of HRZN peptides at peptide concentrations of 100, 10, and 1 µg/mL on defibrinated human red blood cells (hRBCs) with LL-37 used as a control. (**B**) Toxicity assessment of HRZN-15 and polymyxin B using *Galleria mellonella*. Each worm was injected with 10 µg of either HRZN-15 or polymyxin B in 10 µL. Worms injected with 10 µL of PBS were used as controls. The survival of worms was monitored for 3 days. The Mantel–Cox test was performed to calculate statistical significance (* *p* < 0.05, ns = not significant).

After establishing the antibacterial and antibiofilm activities, we sought to test the toxicity of HRZN peptides by challenging defibrinated human red blood cells with the peptides for 1 h at 37 °C (Figure 3A). Defibrinated blood was chosen over EDTA-treated blood because we observed high hemolysis results when using the latter that did not correlate with the published results [69]. All peptides showed a high degree of hemolysis, except HRZN-13 and -14. 

Hemolysis was detected by the measurement of hemoglobin released (OD_540_). The percentage of hemolysis was calculated relative to the Triton X-100 control (100%) control. HRZN-13 and -14 showed negligible hemolysis (0.7% and 5.6%) with incubation at a 100 µg/mL concentration of peptide. The hemolysis activity of these two peptides is less than LL-37, supporting our conclusion of little to no toxicity of the peptides. HRZN-15 and -16 completely lysed the human red blood cells at 100 µg/mL, whereas HRZN-17 lysed 74% of the hRBCs. At 10 µg/mL, HRZN-16 demonstrated 67% hemolysis, followed by HRZN-17 and HRZN-15 showing 18% and 8.6% hemolysis. These hemolysis values are higher than LL-37, demonstrating that HRZN-15, -16, and -17 have high levels of toxicity. All peptides presented little to no hemolysis at 1 µg/mL. The human cathelicidin peptide LL-37 showed 5.4% and 22% hemolysis at 10 and 100 µg/mL. A similar hemolysis of LL-37 has also been reported at 25 µM (~112 µg/mL) [70]. 

The overall toxicity of the most active peptide HRZN-15 was then assessed in vivo in the waxworm *Galleria mellonellla* model. Although animal models are the most relevant for the assessment of novel therapeutics against MDR *A. baumannii* infection [58,59,71], the waxworm has been developed as a rapid first assay against *A. baumannii* infection [72,73,74,75,76,77,78,79]. We first had to assess the toxicity of HRZN-15 in the waxworm. Waxworms were injected with 10 µg of HRZN-15 or polymyxin B in 10 µL of PBS, and survival was recorded every 24 h for 72 h. HRZN-15-treated worms resulted in only 40% survival of worms (*p* < 0.05) (Figure 3B). This result is consistent with the HRZN-15 hemolysis results. Worms injected with polymyxin B showed 80% survival, and the result was not statistically different from the PBS-injected group (90% survival). Thus, HRZN-15 was toxic by these assays, and the peptide needs further development to reduce its toxicity. Because of this high level of toxicity of the peptide itself, we were unable to proceed to test the ability of HRZN-15 to eradicate an MDR *A. baumannii* infection in the waxworm model. 

## 3. Discussion

As multidrug-resistant wound infections continue to be a danger to both civilians and military personnel, there is an urgent need to discover new antimicrobials to target these bacteria, especially MDR *A. baumannii.* AMPs represent a new class of antimicrobials that have rapid killing activity, different mechanisms of action than antibiotics, and a low propensity for resistance acquisition [80]. AMPs with activity against *A. baumannii* have been developed through rational design and other similar approaches [64,81], and we seek to expand the number of active peptides against this organism. 

Computational approaches to designing new antimicrobial peptides are emerging as a useful and productive approach to generating novel peptide candidates with desired or useful properties [39]. We implemented a novel computational analysis method of our input dataset, using DFT developed by Mishra and Wang, 2012 [48], followed by a positional analysis protocol developed by us [40], “DFT plus PA”. We designed our novel peptides (HRZN) by analyzing a dataset of collected peptide sequences with activity against any strain of *A. baumannii*, as this was the peptide property on which we wanted to focus our design efforts. Additional design parameters included designing a helical peptide with a moderate net cationic charge and a favorable hydrophobic index. We produced a small set of new computationally designed HRZN peptides, which were all previously unknown or unidentified and were tested against MDR *A. baumannii* for their antibacterial and antibiofilm activities. 

Our aggressive approach of testing only against MDR *A. baumannii* sets a high bar for the performance of the peptides, but also quickly eliminates any inactive peptides from consideration for our final desired clinical application, which is the treatment of complex wounds infected with MDR *A. baumannii*.

HRZN peptides demonstrated robust antibacterial activity against 3 MDR *A. baumannii* strains, with HRZN-15 exhibiting the most potent activity (MIC 4 µg/mL). In a previous study where we designed new peptides (PHNX), MIC values were higher [40], illustrating the advantage of using this highly curated dataset for analysis. We also added amide groups at the C-terminus of each peptide, as they have been shown to increase the propensity to form α-helix as well as increase bacterial membrane disruption [82,83]. The most active peptide, HRZN-15, showed rapid killing activity in a dose- and time-dependent manner in the MIC range. The peptide exhibits complete killing against two different MDR *A. baumannii* strains within 3 h at MIC levels and within 1 h at higher peptide concentration. Unlike conventional antibiotics, antimicrobial peptides tend to act directly on bacterial membranes [84]. An analysis of the mechanism of action revealed that HRZN-15 mainly interacts with bacterial membranes. The peptide disrupted the membrane causing depolarization and permeabilization effects. Therefore, HRZN-15 is likely to perturb the membrane, causing cell damage and leading to leakage of intracellular constituents, as evident by SEM images. A similar mode of action was previously demonstrated in LL-37, indicating its potential to interact with bacterial membranes [85]. Another extensively studied AMP, magainin 2, was found to induce membrane permeabilization by forming pores on bacterial membranes [86]. 

The HRZN-15 peptide also exhibited significant antibiofilm activity against MDR *A. baumannii* biofilm. The antibiofilm activity correlated with the MIC; thus, the effect is likely due to killing the bacteria in the biofilm rather than inhibiting biofilm production itself. This is a strong positive characteristic of this peptide that can kill bacteria when they are within the biofilm. HRZN-15 was even able to eradicate pre-formed 24 h biofilm from MDR *A. baumannii* biofilm, unlike the other two antibiofilm peptides tested in this study.

Finally, we evaluated the toxicity of the HRZN-15 peptide through hemolysis and an in vivo waxworm model, and unfortunately, HRZN-15 had significant toxicity in both assays. Thus, more development is needed to reduce the host-directed toxicity of HRZN-15 while retaining its very significant anti-*A. baumannii* and antibiofilm activities and low propensity to induce antimicrobial resistance, whether by incorporating HRZN-15 into a mixture with antibiotics while reducing its concentration, which can result in beneficial synergy and lower toxicity by selective drug delivery in focusing on topical applications, or by modifying its sequence [87]. HRZN-15 toxicity also could be improved by amino acid substitution. Since HRZN-13 did not induce hemolysis, it could be used as a template to re-design HRZN-15 because they share ten residues on the same position, leaving three positions for manipulation.

Computational prediction is early in its development and usually involves the application of machine learning to large datasets of antimicrobial peptide sequences [39,88]. Several successful peptides have been generated by this method [88,89,90,91,92,93], but they generally rely on an analysis of the entire dataset of antimicrobial peptide sequences, either in APD or CAMP-R3, for example, to elucidate properties or sequences that may be generally antibacterial and hopefully active against the organism of interest. Our approach was to be more focused on analyzing the peptide sequences with proven activity against any strain of *A. baumannii* in order to design a new peptide. Our study also highlights some of the current challenges of computational prediction in the design of novel AMPs [39]. For example, various toxin and hemolysis predictors scored HRZN-13 and -14 as highly toxic and hemolytic when, in fact, they are not. Future advances in hemolysis and toxicity predictors for peptides could be very helpful in these workflows. 

Another challenge in computational antimicrobial peptide design is the accuracy of antimicrobial activity prediction by the various algorithms. This is balanced in our workflow by considering the outputs of as many different activity predictors as we know that have different algorithms or datasets. For this analysis, we gave every activity predictor an equal “vote” in the final prediction. Overall, the online predictors of the antimicrobial activity of peptides were fairly accurate for the HRZN series of peptides with respect to MIC activity. This contrasts with our previous series of designed peptides (PHNX), in which the online predictor scores correlated with EC_50_ or low-salt buffer antimicrobial activity of peptides rather than MIC or high-salt buffer antimicrobial activity. Given the differences in the starting data for this study (GRAMPA) vs. the prior PHNX study (APD3), the GRAMPA dataset only reports peptides with MIC activity, thus providing a very robust set of input sequences. Of note, the GRAMPA database only referred to *A. baumannii* strains generically, or if the strains were named, they were not predominantly multidrug-resistant. Our results demonstrate that we were able to use these peptides to design novel peptides that are highly active against multidrug-resistant strains of *A. baumannii*. 

Potential novel therapeutics against MDR *A. baumannii* are highly relevant to clinical wound infections, especially on the battlefield [3,8,9]. Future work studying the structures of HRZN peptides in an effort to reduce toxicity could generate active peptides with improved toxicity profiles. 

## 4. Materials and Methods

### 4.1. DFT plus PA Computational Approach to Design New Peptides 

Peptides HRZN 13 to 17 were designed by database filtering technology (DFT) with a positional analysis (PA) filter, as previously described [40,48]. Figure S1 depicts a flowchart of the design of HRZN peptides. Dataset 1 was developed by collecting all sequences with reported activity against *A. baumannii*. The GRAMPA database [43] consists of 6760 unique sequences and 51,345 MIC values against various organisms and was utilized to generate novel peptides. A total of 643 peptides with MIC values against *A. baumannii* were collected (Table S1). By removing duplicate sequences, we generated Dataset 1 (374 sequences, Table S2), and this dataset was used for designing novel HRZN peptides. 

#### 4.1.1. HRZN-13 and -14

Although the normal criteria for “good AMPs” is MIC ≤ 8 µg/mL, an MIC of 16 µg/mL was used as a cut-off where only AMPs with activity ≤ 16 µg/mL were selected to increase the number of AMPs to enable a better design of peptides using the DFT method [94]. After ≤16 µg/mL selection, we assessed the most frequently occurring length of all AMPs within the dataset following the DFT approach. Thus, peptides of 13 residue lengths were the most frequently occurring length to design the new peptides, followed by lengths 12 and 14. Our previously described method called positional analysis (PA) identifies the most frequently occurring AMP per position at the most frequently occurring length (*n* = 12–14 m). To preserve the helical structure of AMPs, which historically reflects their antimicrobial activities [95], prolines were disallowed at all positions [96]. At position 11, both amino acids R and K occurred most frequently and hence, two iterations of the AMPs were generated: HRZN 13 and HRZN 14. 

#### 4.1.2. HRZN-15

The peptide was obtained from Dataset 1 spanning lengths 13 and 14. The 16 µg/mL cut-off was expanded to include peptides with MICs > 16 µg/mL and a comprehensive dataset, as this increases the total number of peptides for the DFT plus PA, thus strengthening the likelihood of PA to work well [40]. Having more data points increases the efficacy of PA to generate better output, and this is what we observed with HRZN-15.

#### 4.1.3. HRZN-16 and -17

As mentioned, our goal was to design helical peptides, thus peptides HRZN-16 and -17 were designed using a pattern as follows: AMPs of lengths 13 ± 3 amino acids and 13 ± 4 amino acids from Dataset 1 were used for positional analysis. Thus, all AMPs from the database of lengths 10–16 were used for positional analysis to generate HRZN-16 and similarly, all AMPs from the GRAMPA database of lengths 9–17 were used to generate HRZN-17. We chose residue numbers ± 3 or ± 4 to include helical peptides because an alpha helix has ~3.6 residues per turn [97], and ± 3 or 4 as a number is used maintain a helical turn.

### 4.2. Peptide Synthesis

Peptides were synthesized by Fmoc solid phase synthesis from ChinaPeptides Inc. (Shanghai, China) with a high purity of ≥ 98%, and LL-37 was obtained from Direct Peptides Inc. with a high purity of 99%. Purity was also confirmed by reverse-phase high-performance liquid chromatography and mass spectrometry. All peptides, except for LL-37, were amidated on the C-terminus to increase the activity and maintain the stability of the helix [83]. 

### 4.3. Bacterial Strains

Four strains of multidrug-resistant (MDR) *A. baumannii* were used in this study (Table 5): BAA-1710, -1794, and-1800, obtained from American Type Culture Collection (Manassas, VA, USA), and AB5075 MRSN 959, obtained as part of the MDR diversity panel from BEI Resources (NR-52248). They have been well-characterized as a model strain for MDR *A. baumannii* for studying pathogenesis and antimicrobial testing [61]. Thus, the AB5075 strain was used in all assays except MBIC. All bacteria were grown in Tryptic Soy Broth (BD 211825) in a shaking incubator overnight at 37 °C following Jacobs and Zurawski [98]. Bacteria were aliquoted and stored at −80 °C with a final glycerol concentration of 20%. Before each assay, bacteria were grown on a Tryptic Soy agar, and three to five colonies with opaque morphology were selected to be grown for experiments. 

### 4.4. Screening Peptides for Antibacterial and Antibiofilm Activities

As the first step, antibacterial screening (*n* = 3) was performed on MDR strains of *A. baumannii* listed in Table 5, with each peptide at a concentration of 100 μg/mL in MHB. Each well was inoculated with 50 µL of 1 × 10^6^ CFU/mL of bacteria, similar to the MIC protocol for AMPs [62]. Growth was measured spectrophotometrically at OD600 nm at 20–24 h, following the CLSI protocol for *A. baumannii* incubation for MIC [99]. Similarly, biofilm formation was first established for tested *A. baumannii* strains using our crystal violet-staining protocol described below. Then, antibiofilm screening (*n* = 3) of each peptide against biofilm formation of *A. baumannii* was determined at 100 μg/mL. Peptides that were active in this screen were then analyzed in more detail as described below. Positive controls were used, such as LL-37 and polymyxin B [18,66,67]. Samples containing only the medium were considered as the sterility control whereas samples containing only bacteria served as the growth control.

### 4.5. Minimum Inhibitory Concentration (MIC)

Minimum inhibitory concentration (*n* = 3) was performed according to the CLSI protocols with a minor modification, in which Difco Mueller Hinton broth (BD 275730, Franklin Lakes, NJ, USA) was used instead of cation-adjusted Mueller Hinton broth following Wiegand et al.’s standardized protocol for testing AMPs [62]. Bacteria were grown overnight in an opaque colony, and the concentration was determined using McFarland’s technique [62]. An aliquot of 50 µL of 1 × 10^6^ CFU/mL of bacteria was incubated with a decreasing series of peptide concentration to achieve a final bacterial concentration of 5 × 10^5^ CFU/mL at 37 °C 5% CO_2_ for 20–24 h in Difco Mueller Hinton broth. Dilutions were performed in a 96-well polypropylene plate (Corning 3879, Corning, NY, USA). This is critical to avoid peptide binding to the plate. Results were obtained by measuring bacterial growth at OD600 nm. LL-37 and polymyxin B were used as positive controls [18,66,67,100]. Samples containing only the medium and medium with peptides were used as the sterility controls and samples containing only bacteria in MHB served as the growth control. Each experiment was performed twice, and one representative experiment is shown.

### 4.6. Time-Kill Kinetics

An aliquot of 50 µL of 1 × 10^6^ CFU/mL of bacteria (*n* = 3) was incubated with 50 µL of 1, 5, and 10 MIC folds of HRZN-15 or polymyxin B for 10, 30, 60, and 180 min in Difco Mueller Hinton broth (BD 275730). At the various timepoints, samples were serially diluted, and CFUs were counted by spot plating on a Tryptic Soy agar [101].

### 4.7. Minimum Biofilm Inhibitory Concentration (MBIC)

The MBIC assay measures the lowest concentration of a compound that inhibits the biofilm formation of an organism [14,15,16,17,18,26,27,28]. A series of decreasing peptide concentrations starting from 64 μg/mL to 0.25 μg/mL (100 µL) were incubated with 1 × 10^5^ CFU of *A. baumannii* in 100 µL of Tryptic Soy broth (TSB) in a polystyrene 96-well plate (Falcon 353072, Corning, NY, USA) for 24 h at 37 °C to assess biofilm formation inhibition, as described previously with minor modifications [18]. After the incubation of bacteria with AMPs, OD600 nm was measured for approximate bacterial growth. Media were carefully removed, and wells (*n* = 3) were rinsed with tap water. Plates were heat-fixed at 70 °C for 1 h, and 200 µL of 0.1% crystal violet diluted in deionized water were added to wells and allowed to incubate for 15 min. Excess stain was rinsed off with tap water, and the plates were air-dried. After that, 200 µL of 33% glacial acetic acid was added to resolubilize the stain, and biofilm mass was measured by a spectrophotometric reading at OD_590_. Bacteria alone (no treatment) served as the 100% biofilm control, and media alone and media with a peptide/drug were used as the sterility control and the 0% biofilm control. IDR-1018, LL-37, and polymyxin B were used as the positive controls [18,66,67,102].

### 4.8. Minimum Biofilm Eradication Concentration (MBEC)

The MBEC assay measures the lowest concentration of an antimicrobial agent that can eradicate established biofilms. Using the MBEC assay in the Biofilm Inoculator 96-well base (Innovotech Cat #1911, Edmonton, AB, Canada) and following the manufacturer’s protocol [103], 150 µL (*n* = 6) of 1 × 10^6^ CFU/mL of AB5075 in TSB was incubated for 24 h at 37 °C at 110 rpm for biofilm formation [104]. The lid containing pegs was rinsed with sterile PBS for 5 min, and then the lid was placed in a peptide challenge polypropylene 96-well plate containing a decreasing series of drug concentrations and incubated for 24 h at 37 °C at 110 rpm. Pegs were washed three times with tap water, heat-fixed for 70 °C for 1 h, and stained with 0.1% crystal violet for 15 min. Excess stain was rinsed off with tap water and allowed to dry. Stained pegs were resolubilized with 200 µL of 33% glacial acetic acid. OD_590_ was obtained to measure the remaining biofilm. Pegs with only bacteria served as the growth control and 100% biofilm control. Media alone and media with a peptide/drug served as the sterility controls. LL-37, IDR-1018, and polymyxin B were used as the positive controls [18,66,67]. The experiment was performed twice.

### 4.9. Resistance Induction

Sequential passaging of AB5075 with MIC levels of HRZN-15 and colistin to achieve final peptide concentrations of 4 and 0.5 µg/mL, respectively, in Difco MHB (BD 275730) was performed for 15 days following Mourtada et al. 2019 [50]. Bacteria were grown in 1 mL of MHB for 24 h. Experimental tubes that show growth in the presence of peptide/drug concentration were diluted 1:100. MIC was determined for HRZN-15 and colistin was determined for every five passages using the MIC method mentioned previously. The concentration of added peptide/drug was increased as resistance was observed. Colistin is reported to induce resistance within approximately 5–7 days for *A. baumannii* [50].

### 4.10. Membrane Permeabilization Assay

Membrane permeabilization was assessed using ethidium bromide uptake after peptide exposure, as we have previously described [14,15,16,17,18,26,27,28]. AB5075 was grown on a Tryptic Soy agar for 18–24 h. Colonies presenting opaque morphology were picked and resuspended in DPBS (Gibco, Waltham, MA, USA) to achieve 0.1 OD_600_. A total of 180 µL of the bacterial suspension was mixed with a final peptide concentration of 50 µg/mL and a final ethidium bromide concentration of 10 µM in a black 96-well flat plate (Ultracruz Polypropylene Microplate sc-204462, Santacruz, CA, USA). The plate was transferred immediately to BioTek Cytation 5 (Santa Clara, CA, USA) for fluorescence intensity readings every 2 min for 20 min at 37 °C (excitation: 535 nm; emission: 590 nm). The relative fluorescence units (RFUs) were calculated. Tested samples minus no peptide control that has EtBr. LL-37 and polymyxin B were used as the controls in this experiment. The experiment was run in three replicates twice. Student’s t-test was used to determine statistical significance.

### 4.11. Membrane Depolarization Assay

A membrane depolarization assay was performed using a cationic dye, 3,3′-dipropylthiadicarbocyanine iodide (DiSC_3_(5)) [105] as we previously described, with some modifications [14,16,17,27]. Briefly, AB5075 was grown overnight on a Tryptic Soy agar, and opaque colonies were emulsified in DPBS (Gibco) until the 0.5 McFarland standard was achieved (~1 × 10^8^ CFU/mL). A bacterial suspension of 4 × 10^7^ CFU/mL was prepared and washed twice with DPBS, and then bacteria were resuspended in DPBS containing 10 µg/mL of DiSC_3_(5). An aliquot of 100 µL of the bacteria DiSC_3_(5) suspension was added to a black 96-well flat plate (Ultracruz Polypropylene Microplate sc-204462, Santacruz, CA, USA). The plate was incubated at room temperature in BioTek Cytation 5 and monitored until fluorescence stopped decreasing. An aliquot of 100 µL of peptide or DPBS (the untreated control) was added to each well (a final concentration of peptide of 50 µg/mL) and the plate was immediately returned to the plate reader. The peptide concentration was chosen because the peptide did not kill bacteria within 30 min at 40 µg/mL, as seen in Figure 2A. Readings were taken every 15 s for 20 min (excitation = 622 nm; emission = 670 nm). The experiment was repeated twice.

### 4.12. Scanning Electron Microscopy 

Visualization of bacteria was performed as previously described [106] but with a different fixation method. Briefly, AB5075 was grown on TSA, and three to five colonies with opaque morphology were resuspended in DBSP (Gibco). The cells were washed twice with DPBS and resuspended to achieve a bacterial concentration of 2 × 10^8^ CFU/mL. Using a polypropylene (Corning 3879) 96-well plate, 50 µL of the bacteria was mixed with 50 µL of HRZN-15 (100 µg/mL) to yield final concentrations of 1 × 10^8^ CFU/mL and 50 µg/mL, respectively. The plate was incubated at room temperature for 20 min. The samples containing bacteria only or bacteria and HRZN-15 were filtered through a 0.1 µm polycarbonate filter (Whatman 10419504) after treating the membrane with 0.1% poly-L-lysine to improve bacterial adhesion [107]. Instead of using chemical fixation, the flash-freezing method was used, as described previously [108]. Membranes containing samples were placed in 10 mL beakers (VWR 10754-696) and subjected to liquid nitrogen for 20 min. Then, samples were freeze-dried for 24 h. Membranes were mounted on a double carbon tape that was attached to a specimen mount. The membranes were sputter coated and visualized using field emission scanning electron microscope JSM-7200F (JEOL USA, Inc., Peabody, MA, USA) at 15 kV or as indicated.

### 4.13. Hemolysis

To measure the hemolytic activity of peptides, 2% human red blood cells were added to various dilutions of peptide reconstituted in PBS in a sterile U-bottom polypropylene 96-well plate following a protocol developed in our laboratory Briefly, defibrinated human blood was obtained from de-identified healthy donors from BioIVT (Westbury, NY, USA). An aliquot (1 mL) of blood was centrifuged at 1600× *g* for 10 min and plasma was discarded, and then 1 mL of sterile 1× PBS (HyClone) was used to wash red blood cells at least three times. In the last wash step, the supernatant was discarded, and the pallet was resuspended with 750 µL of sterile PBS. RBC suspension (2% RBC) was prepared by adding 200 µL of washed RBCs to 9.8 mL of sterile 1× PBS. A total of 50 µL of 2% RBCs were added to each well (*n* = 3) containing diluted peptides, resulting in a final peptide concentration of 100, 10, and 1 μg/mL. A total of 2% RBCs with 1× PBS alone served as the negative control (no peptide), and 2% RBC in Triton X-100 served as the positive control. The plate was incubated for 1 h at 37 °C and then centrifuged at 1000 rpm for 2 min. The supernatant was transferred to a fresh 96-well plate (tissue culture-treated Falcon 353072) and read at OD540. The percentage of hemolysis was calculated based on the 100% hemolysis control (Triton X-100). The experiment was repeated twice.

### 4.14. Waxworm Toxicity Testing

*Galleria mellonella* were obtained from Vanderhorst Wholesale (Saint Mary’s, OH, USA), and 10 worms with equal weights between 200 to 300 mg were assigned to each group. Each worm was injected with 10 µL of PBS containing 10 µg of HRZN-15 or polymyxin B in the worms’ last right proleg using a 250 µL Hamilton syringe with a 30G needle (Hamilton), and the survival of worms was assessed for 72 h, as previously described [18,109]. The experiment was performed twice, and the results were analyzed by the Mantel–Cox test.

## 5. Patents

The peptide design method(s), particularly the positional analysis, described in this manuscript are part of a patent application filed by George Mason University (USPTO application number 17/822,161).

## Figures and Tables

**Table 1:** Designed HRZN peptides and predicted features. In helical wheel projections, blue, red and purple represent polar residues and yellow, green and grey represent nonpolar residues. In the predicted structures (helices), the colors have no meaning.

Peptide	Sequence	Charge	% Hydrophobicity (Hydrophobic Moment)	Predicted Structure AlphaFold2	Helical Wheel
HRZN-13	FLWRISKFLGKKL-NH_2_	+5	54% (0.537)	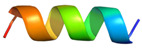	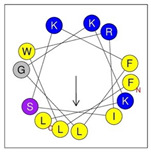
HRZN-14	FLWRISKFLGRKL-NH_2_	+5	54% (0.539)	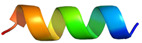	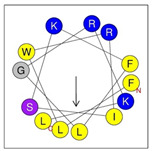
HRZN-15	FLPWISKFLGKIL-NH_2_	+3	62% (0.659)	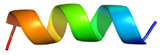	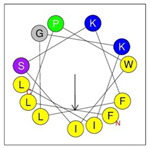
HRZN-16	FLKKIWKLLGKLL-NH_2_	+5	62% (0.877)	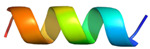	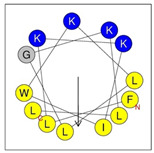
HRZN-17	KLWKLLKKLGRLL-NH_2_	+6	54% (0.804)	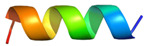	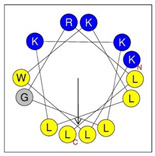
IDR-1018	VRLIVAVRIWRR-NH_2_	+5	67% (0.271)	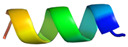	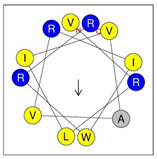
LL-37	LLGDFFRKSKEKIGKEFKRIVQRIKDFLRNLVPRTES	+6	35% (0.521)	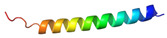	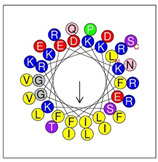

**Table 2:** Antimicrobial activity prediction results. Predictions that are lower than expected are bolded.

Name	Ferguson	CAMPR3				ClassAMP		DBAASP	PepVAE3
	SVM	SVM	RF	ANN	DA	SVM	RF		
HRZN-13	1	1.00	1.00	AMP	0.99	0.99	1.00	AMP	AMP
HRZN-14	1	0.99	0.99	AMP	0.99	0.99	1.00	AMP	AMP
HRZN-15	0.98	0.96	0.99	AMP	1.00	0.99	1.00	AMP	AMP
HRZN-16	1	1.00	0.98	AMP	1.00	0.96	0.99	AMP	AMP
HRZN-17	1	0.93	**0.60**	AMP	0.99	0.94	0.96	AMP	AMP
IDR-1018	**0.15**	0.99	0.97	AMP	0.99	0.97	0.97	Non-AMP	AMP
LL-37	1	**0.76**	**0.75**	AMP	**0.77**	0.97	0.95	AMP	AMP

**Table 3:** The MIC of HRZN peptides against three multidrug-resistant strains of *A. baumannii:* AB5075, BAA-1710, BAA-1794, and BAA-1800.

Peptide/Organism	*A. baumannii* AB5075 (MRSN959)	*A. baumannii* BAA-1710	*A. baumannii* BAA-1794	*A. baumannii* BAA-1800
HRZN-13	32	32	64	64
HRZN-14	16	32	64	32
HRZN-15	4–8	4	4	4
HRZN-16	16	16	16	16
HRZN-17	32	32	32	32
LL-37	8	32	16–32	8
Polymyxin B	0.25–0.5	0.5	0.5	0.5

**Table 4:** MBIC_100_ of *A. baumannii* BAA-1800 and MBEC of AB5075 against tested peptides.

Peptide	MBIC_100_ (µg/mL)	MBEC (µg/mL)
HRZN-15	8	16
LL-37	64	~32
IDR-1018	>64 *	>32
Polymyxin B	1	* 2

* See the discussion of polymyxin and IDR1018 results in the text.

**Table 5:** List of *A. baumannii* MDR strains tested.

Strain	Source	Source Information
AB5075 (MRSN 959)	BEI Resources	Human tibia/osteomyelitis
BAA-1710	ATCC	Human blood
BAA-1794	ATCC	Human sputum
BAA-1800	ATCC	Human deep trachea

## Data Availability

All data used in this study are included in the Supplemental Tables.

## Supplementary Materials

The following supporting information can be downloaded at https://www.mdpi.com/article/10.3390/antibiotics12091396/s1, Figure S1: Flowchart of the DFT plus positional analysis (PA) method used to design HRZN peptides; Figure S2: Minimum inhibitory concentration of peptides against *A. baumannii* AB5075; Figure S3: Minimum inhibitory concentration of peptides against *A. baumannii* BAA-1710; Figure S4: Minimum inhibitory concentration of peptides against *A. baumannii* BAA-1794; Figure S5: Minimum inhibitory concentration of peptides against *A. baumannii* BAA-1800; Figure S6: Minimum biofilm inhibition concentration (MBIC) of HRZN-15, LL-37, IDR-1018, and polymyxin B against *A. baumannii* BAA-1800; Figure S7: Minimum biofilm inhibition concentration (MBIC) of HRZN-15, LL-37, IDR-1018, and polymyxin B against *A. baumannii* BAA-1800 (growth and biofilm); Figure S8: Minimum biofilm eradication concentration (MBEC) of HRZN-15, LL-37, IDR-1018, and polymyxin B against AB5075; Figure S9: Kinetics of membrane depolarization (DiSC_3_(5)) and disruption (EtBr) of HRZN-15, LL-37 and polymyxin B against AB5075 upon exposure to 50 µg/mL of each compound; Figure S10: MIC of passages in in vitro resistance acquisition induction of AB5075 upon exposure to HRZN-15 and colistin for 15 days; Table S1: *A. baumannii*-active peptides from the GRAMPA database; Table S2: Dataset 1 containing unique antimicrobial peptides with activity against *A. baumannii*.
