# Supplementary material for: Computationally Designed AMPs with Antibacterial and Antibiofilm Activity against MDR Acinetobacter baumannii
Source: Antibiotics (Basel). 2023 Sep 1;12(9):1396. doi: 10.3390/antibiotics12091396 (PMC10525135; doi:10.3390/antibiotics12091396)
Supplement: Supplementary file 1 [file antibiotics-12-01396-s001.zip › antibiotics-2557938-SI.pdf]

Supplemental Materials:

Supplemental materials for "Computationally Designed AMPs with Antibacterial and Antibiofilm Activity against MDR Acinetobacter baumannii" By Fahad M. Alsaab, Scott N. Dean, Shravani Bobde, Gabriel G. Ascoli and Monique L. van Hoek

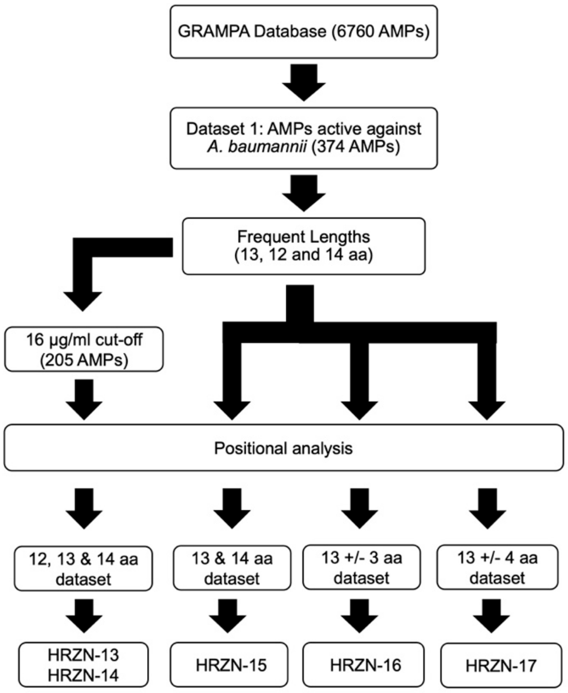

Figure S1. Flowchart of DFT plus positional analysis (PA) method used to design HRZN peptides.

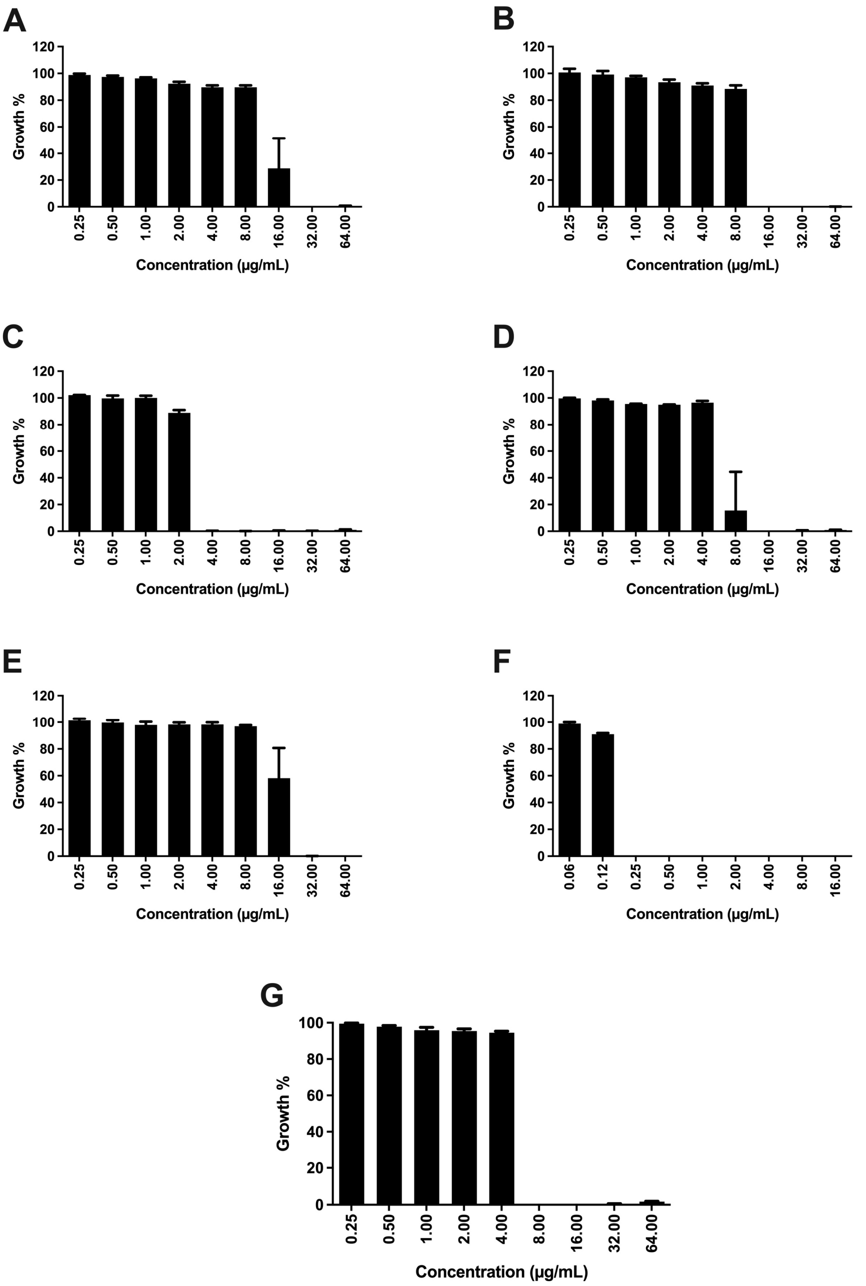

Figure S2. Minimum inhibitory concentration (MIC) of peptides against *A. baumannii* AB5075. (**A**) HRZN-13, (**B**) HRZN-14, (**C**) HRZN-15, (**D**) HRZN-16, (**E**) HRZN-17, (**F**) polymyxin B and (**G**) LL-37 resulted in MIC of 32, 32, 4, 16, 32, 0.5 and 8 μg/mL, respectively.

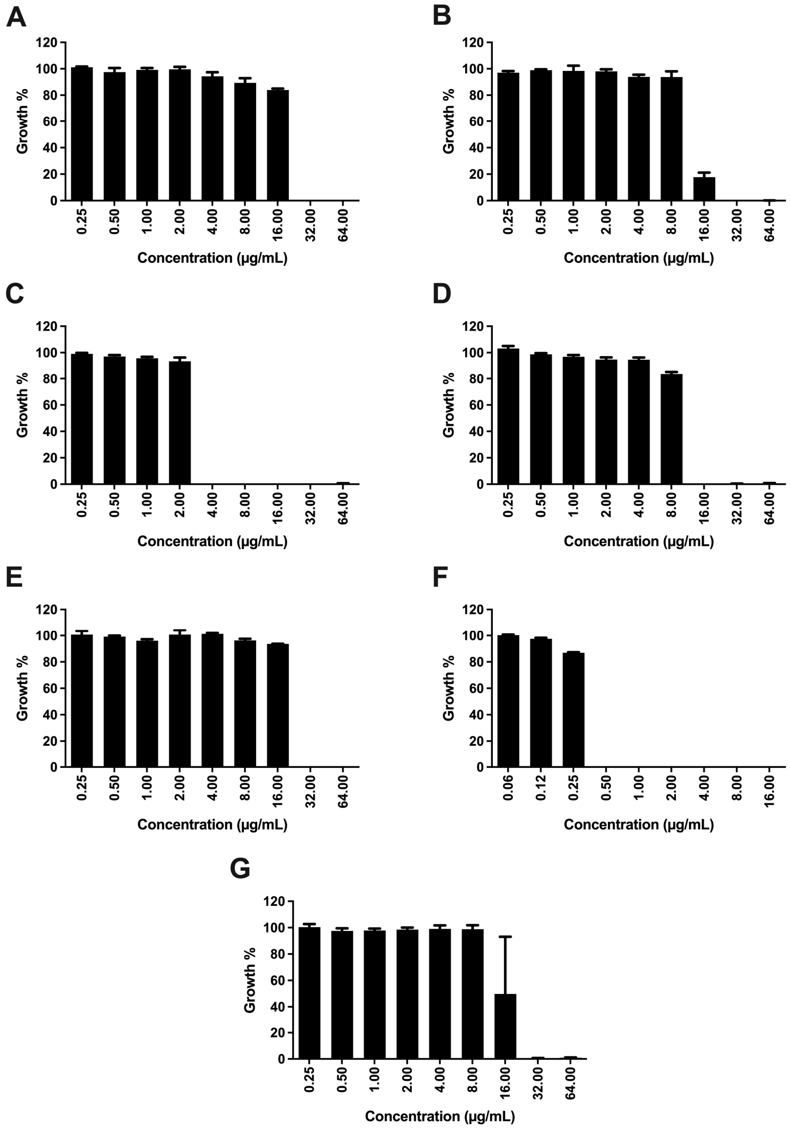

Figure S3. Minimum inhibitory concentration of peptides against *A. baumannii* BAA-1710. (**A**) HRZN-13, (**B**) HRZN-14, (**C**) HRZN-15, (**D**) HRZN-16, (**E**) HRZN-17, (**F**) polymyxin B and (**G**) LL-37 resulted in MIC of 32, 32, 4, 16, 32, 0.5 and 32 µg/mL, respectively.

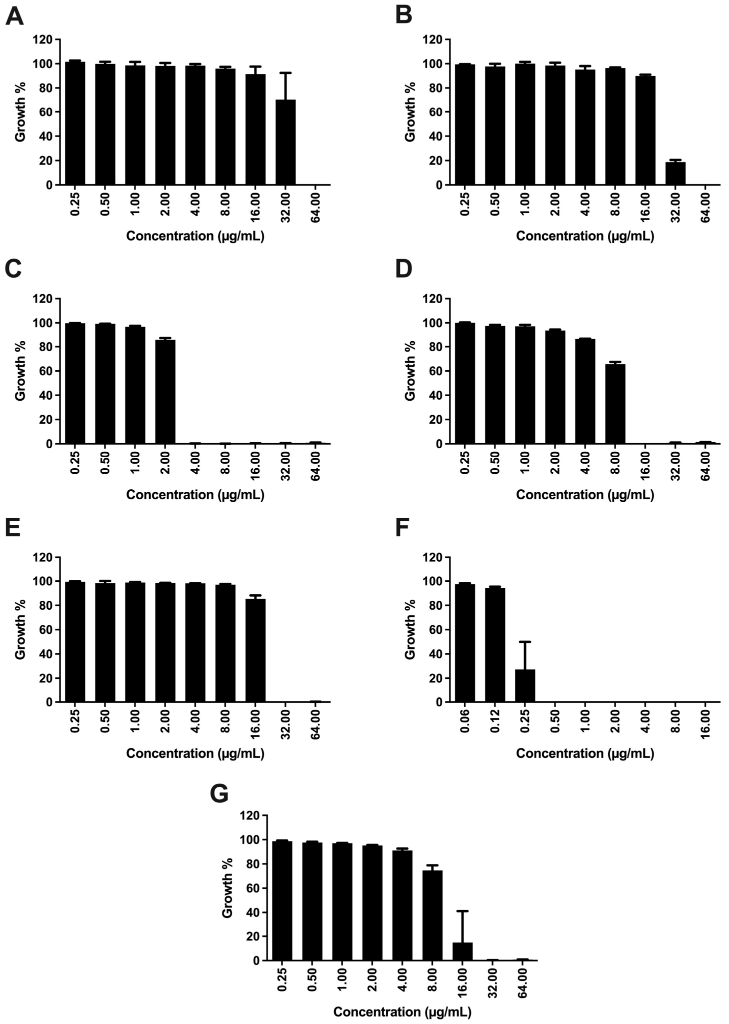

Figure S4. Minimum inhibitory concentration of peptides against *A. baumannii* BAA-1794. (**A**) HRZN-13, (**B**) HRZN-14, (**C**) HRZN-15, (**D**) HRZN-16, (**E**) HRZN-17, (**F**) polymyxin B and (**G**) LL-37 resulted in MIC of 64, 64, 4, 16, 32, 0.5 and 32 μg/mL, respectively.

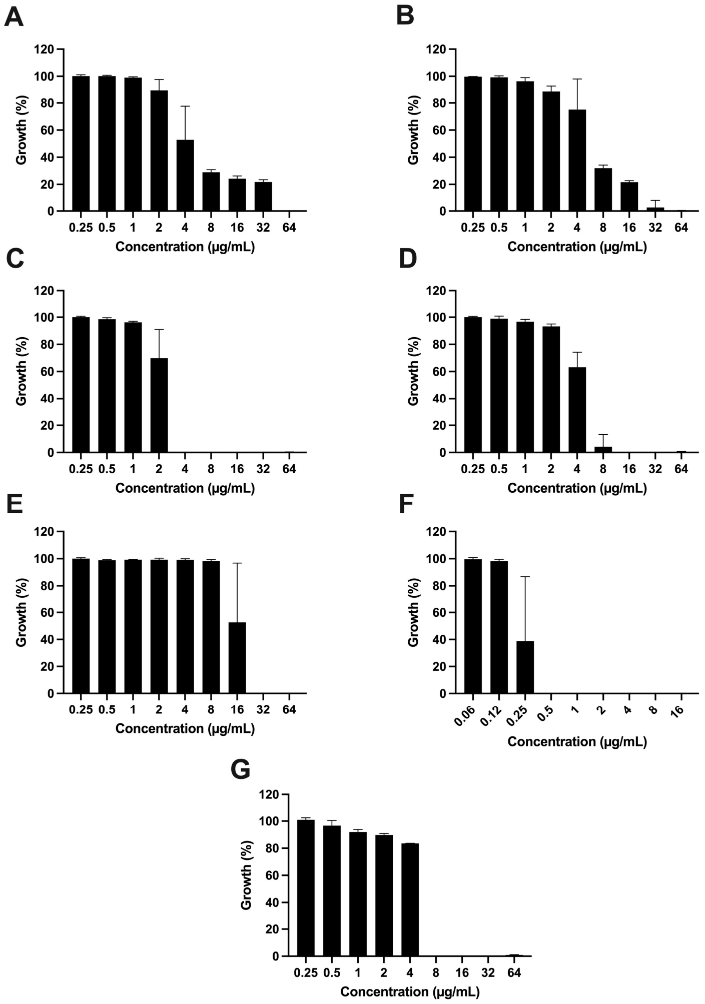

Figure S5. Minimum inhibitory concentration of peptides against *A. baumannii* BAA-1800. (**A**) HRZN-13, (**B**) HRZN-14, (**C**) HRZN-15, (**D**) HRZN-16, (**E**) HRZN-17, (**F**) polymyxin B and (**G**) LL-37 resulted in MIC of 64, 64, 4, 16, 32, 0.5 and 8 µg/mL, respectively.

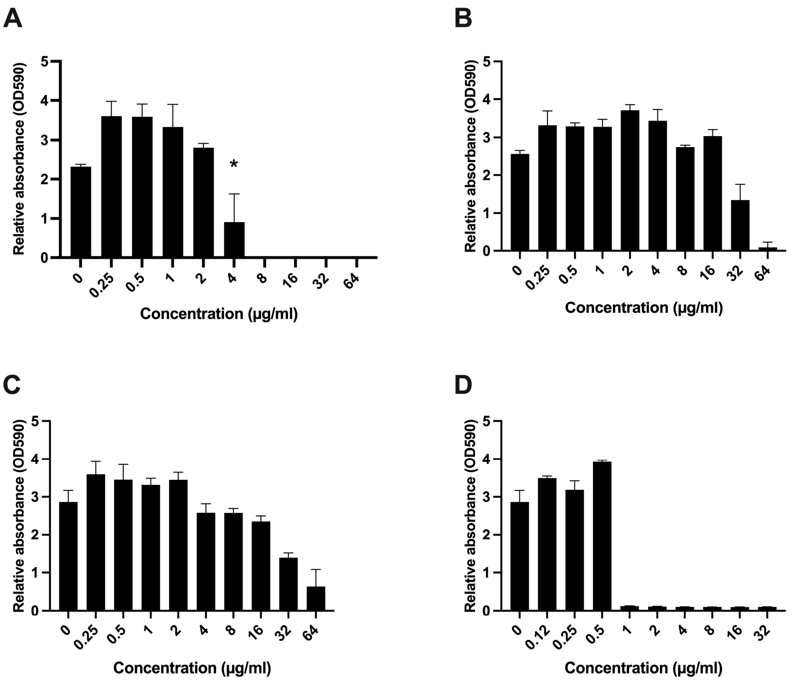

Figure S6. Minimum biofilm inhibition concentration (MBIC) of (**A**) HRZN-15, (**B**) LL-37, (**C**) IDR-1018 and (**D**) polymyxin B against *A. baumannii* BAA-1800. Biofilm detection on a polystyrene 96-well plate at 37°C after 24 h of growth in TSB was detected as the absorbance of crystal violet stain (590 nm). Percent biofilm production is indicated (n = 3), relative to "0 peptide/antibiotic" control. Asterisk * indicates P <0.05 (unpaired t-test).

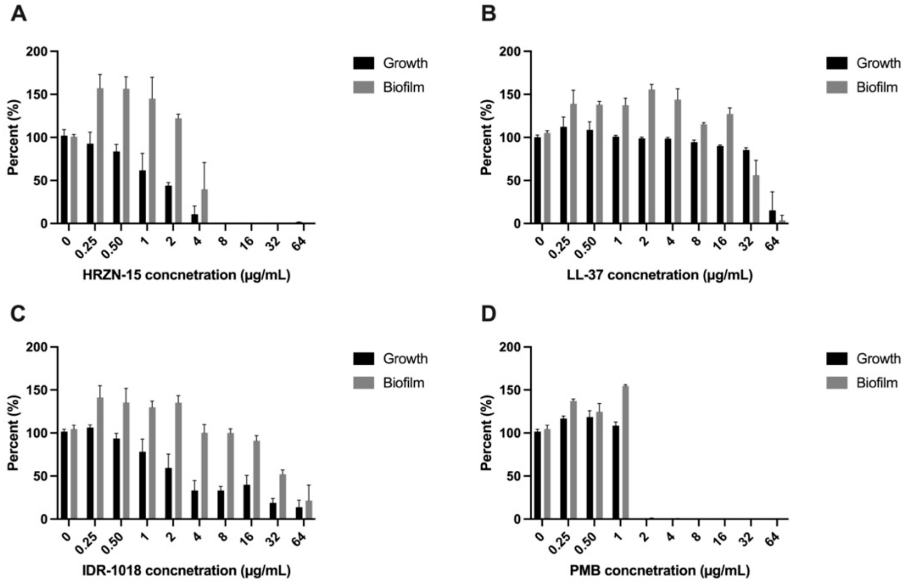

Figure S7. Minimum biofilm inhibition concentration (MBIC) of (**A**) HRZN-15, (**B**) LL-37, (**C**) IDR-1018 and (**D**) polymyxin B against *A. baumannii* BAA-1800. Panels indicate bacterial growth (absorbance at 600 nm) represented in black bars with "0 peptide/antibiotic" control set to 100%. Biofilm detection on a polystyrene 96-well plate at 37°C after 24 h of growth in TSB was detected as the absorbance of crystal violet stain (590 nm). Percent biofilm production is indicated by gray bars (n = 3), relative to "0 peptide/antibiotic" control.

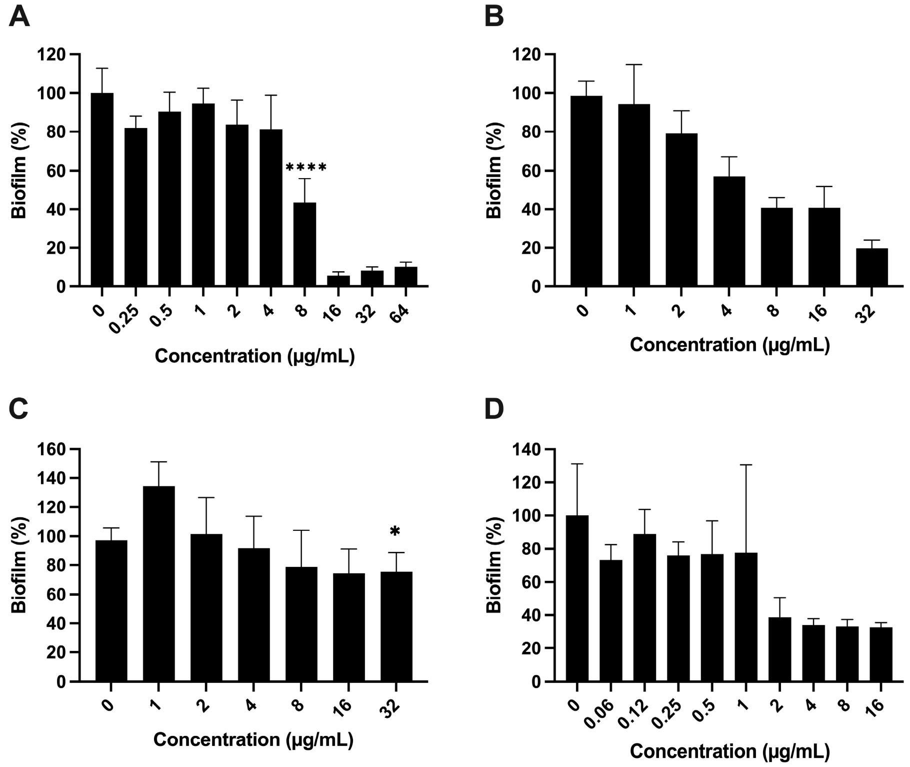

Figure S8. Minimum biofilm eradication concentration (MBEC) of (**A**) HRZN-15, (**B**) LL-37, (**C**) IDR-1018 and (**D**) polymyxin B against AB5075 (n = 6). Biofilm mass on pegs was detected at the absorbance of crystal violet stain (590 nm). Percentage biofilm mass is calculated relative to "0 peptide/antibiotic" control. The experiment was performed twice. (* P <0.05 and **** P <0.0001).

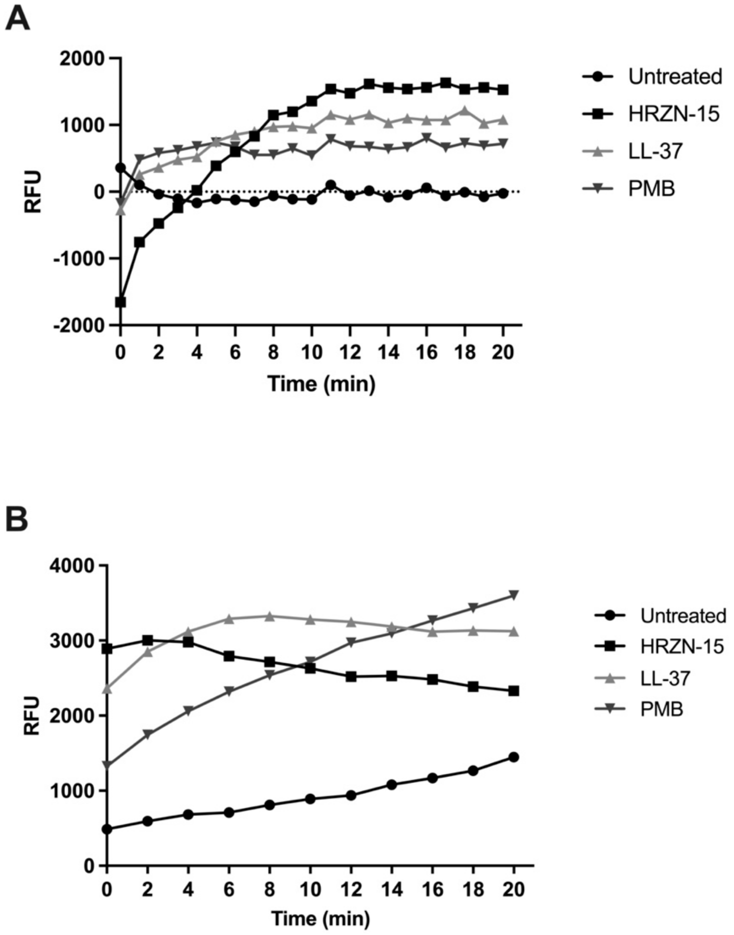

Figure S9. Kinetics of (**A**) membrane depolarization ($DiSC_3(5)$) and (**B**) disruption (EtBr) of HRZN-15, LL-37 and polymyxin B (PMB) against AB5075 upon exposure of 50 μg/mL of each compound.

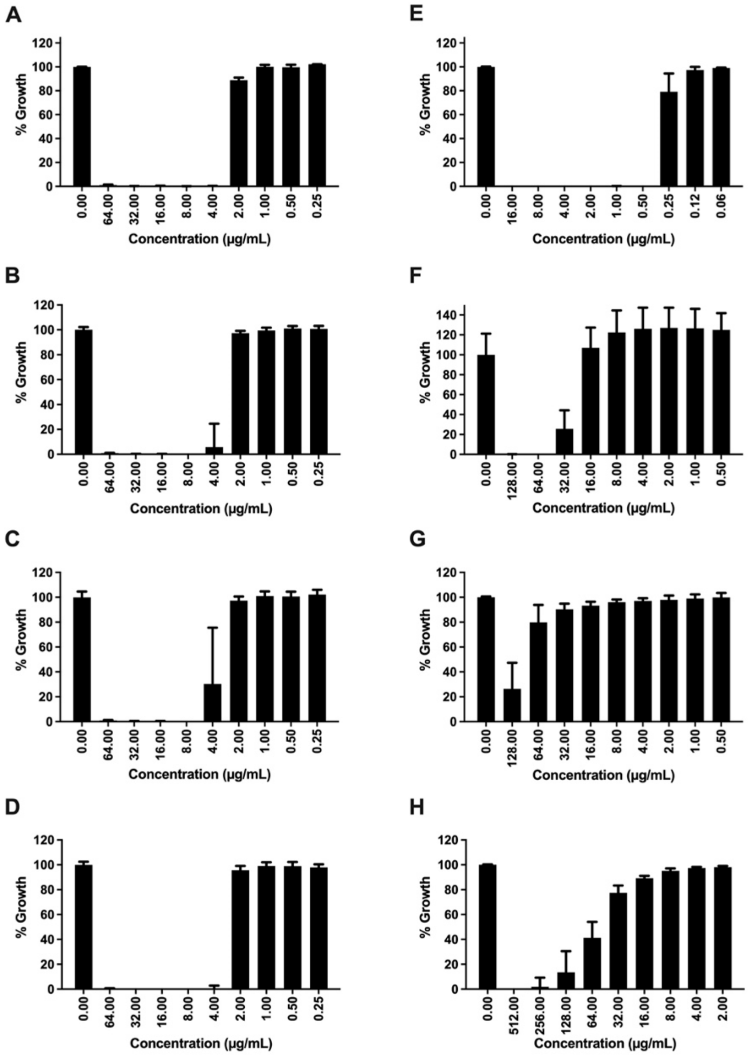

Figure S10. *In vitro* resistance acquisition induction of AB5075 upon exposure of HRZN-15 and colistin for 15 days. (**A** – **D**) shows consistent MIC values of AB5075 exposed to HRZN-15 on passage 0, 5, 10 and 15 (from top to bottom). (**E** – **H**) represents MIC of colistin against colistin-exposed bacteria performed on passage 0, 5, 10 and 15.

Table S1. *A. baumannii*-active peptides from GRAMPA database [43].

Strain	Sequence	µM	µg/mL
ATCC 19606	AAYLLAKINLKALAALAKKIL	3.6	8.0
NA	AAYLLAKINLKALAALAKKIL	7.2	16.0
CCARM 12036	AFHHIFRGIVHVGKTIHRLVTG	2.0	5.0
CCARM 12005	AFHHIFRGIVHVGKTIHRLVTG	2.0	5.0
CCARM 12035	AFHHIFRGIVHVGKTIHRLVTG	2.0	5.0
CCARM 12037	AFHHIFRGIVHVGKTIHRLVTG	2.0	5.0
ATCC 19606	AGRGKQGGKVRAKAKTRSSRAGLQFPVGRVHRLLRKGNY	60.1	256.0

ATCC 19606	AGYLLGKINLKALAALAKKIL	3.7	8.0
NA	AGYLLGKINLKALAALAKKIL	3.7	8.0
ATCC 19606	AGYLLGKINLKPLAALAKKIL	3.6	8.0
NA	AGYLLGKINLKPLAALAKKIL	3.6	8.0
ATCC 19606	AGYLLPKINLKPLAKLPKKIL	54.9	128.0
NA	AGYLLPKINLKPLAKLPKKIL	54.9	128.0
NA	AKKVFKRLGIGKFLHSAKKF	3.1	7.2
KCTC 2508	AKKVFKRLGIGKFLHSAKKF	6.3	14.4
NA	AKRHHGYKRKFH	81.8	128.0
NA	ALASLLKTLSKAAKKALKTLLKALSA	1.4	3.7
ATCC 19606	ALASLLKTLSKAAKKALKTLLKALSA	2.9	7.7
M89952	ALASLLKTLSKAAKKALKTLLKALSA	2.9	7.7
ATCC 17978	ALASLLKTLSKAAKKALKTLLKALSA	5.8	15.4
M89941	ALASLLKTLSKAAKKALKTLLKALSA	5.8	15.4
ATCC 19606	ALASLLKTLSKAKKKKLKTLLKALSA	0.4	1.1
ATCC 17978	ALASLLKTLSKAKKKKLKTLLKALSA	0.4	1.1
M89941	ALASLLKTLSKAKKKKLKTLLKALSA	0.4	1.1
M89952	ALASLLKTLSKAKKKKLKTLLKALSA	0.7	1.9
NA	ALASLLKTLSKAKKKKLKTLLKALSA	0.7	1.9
M89955	ALKSLLATLSKAAKKALKTLLAALSK	0.7	1.9
NA	ALKSLLATLSKAAKKALKTLLAALSK	0.7	1.9
M89953	ALKSLLATLSKAAKKALKTLLAALSK	1.4	3.7
ATCC 19606	ALKSLLATLSKAAKKALKTLLAALSK	2.9	7.7
ATCC 17978	ALKSLLATLSKAAKKALKTLLAALSK	2.9	7.7
M89941	ALKSLLATLSKAAKKALKTLLAALSK	2.9	7.7
ATCC 19606	ALKSLLATLSKAKKKKLKTLLAALSK	0.4	1.1
ATCC 17978	ALKSLLATLSKAKKKKLKTLLAALSK	0.4	1.1
M89941	ALKSLLATLSKAKKKKLKTLLAALSK	0.4	1.1
M89952	ALKSLLATLSKAKKKKLKTLLAALSK	0.7	1.9
NA	ALKSLLATLSKAKKKKLKTLLAALSK	1.4	3.9
M89955	ALKSLLKTLSAAAKKALATLLKALSK	0.4	1.1
M89953	ALKSLLKTLSAAAKKALATLLKALSK	0.7	1.9
NA	ALKSLLKTLSAAAKKALATLLKALSK	1.0	2.7
ATCC 19606	ALKSLLKTLSAAAKKALATLLKALSK	1.4	3.7
ATCC 17978	ALKSLLKTLSAAAKKALATLLKALSK	1.4	3.7
M89941	ALKSLLKTLSAAAKKALATLLKALSK	1.4	3.7
M89963	ALKSLLKTLSAAAKKALATLLKALSK	2.9	7.7
ATCC 17978	ALKSLLKTLSAAKKKKLATLLKALSK	0.2	0.6
ATCC 19606	ALKSLLKTLSAAKKKKLATLLKALSK	0.4	1.1

M89941	ALKSLLKTLSAAKKKKLATLLKALSK	0.4	1.1
NA	ALKSLLKTLSAAKKKKLATLLKALSK	1.4	3.9
ATCC 17978	ALKSLLKTLSKAAAAALKTLLKALSK	2.9	7.7
M89941	ALKSLLKTLSKAAAAALKTLLKALSK	2.9	7.7
ATCC 19606	ALKSLLKTLSKAAAAALKTLLKALSK	5.8	15.4
M89963	ALKSLLKTLSKAAAAALKTLLKALSK	5.8	15.4
NA	ALKSLLKTLSKAAAAALKTLLKALSK	92.8	246.2
ATCC 19606	ALWHHLLHHLLHSAHHLG	15.0	31.9
ATCC 19606	ALWKKLLKKLLKSAKKLG	1.9	3.9
ATCC 19606	ALWMTLKKKVLKAAAKALNAVLVGANA	0.4	1.1
ATCC 17978	ALWMTLKKKVLKAAAKALNAVLVGANA	0.7	2.0
NA	ALWMTLKKKVLKAAAKALNAVLVGANA	1.4	3.9
NA	ALWMTLLKKVLKAAAKALNAVLVGANA	0.7	1.9
ATCC 19606	ALWMTLLKKVLKAAAKALNAVLVGANA	2.8	7.8
ATCC 17978	ALWMTLLKKVLKAAAKALNAVLVGANA	2.8	7.8
ATCC 19606	ALWRRLLRRLLRSARRLG	3.8	8.5
NA	AMVGT	17.1	8.2
NA	AMVSS	15.2	7.5
ATCC 15308	AQWFAIQHISLNPPRSTIAMRAINNYRWR	1.5	5.3
ATCC 15308	AQWFAIQHISLNPPRSTIAMRAINNYRWRSKNQNTFLR	0.9	4.0
ATCC 19606	AVAGEKLWLLPHLLKMLLTPTP	163.8	400.0
Q12	AWRWKAFRNCWRVRSSSL	13.9	32.0
Q13	AWRWKAFRNCWRVRSSSL	27.7	64.0
NA	CLRKLRKRLLC	22.8	32.0
NA	CYCRIPACIAGERRYGTCIYQGRLWAFCC	0.3	1.0
CIP 70	CYCRIPACIAGERRYGTCIYQGRLWAFCC	1.2	4.0
NA	DCYCRIPACIAGERRYGTCIYQGRLWAFCC	0.3	1.0
CIP 70	DCYCRIPACIAGERRYGTCIYQGRLWAFCC	1.1	4.0
ATCC 19606	DDALHHLLHHLLHHL	100.0	182.1
ATCC 19606	DDALKHLLKHLLKHL	50.0	89.7
ATCC 19606	DDALKKLLKKLLKKL	25.0	44.2
ATCC 19606	DDALRHLLRHLLRHL	100.0	187.8
ATCC 19606	DDALRRLLRRLLRRL	100.0	193.5
ATCC 19606	DHYNCVSSGGQCLYSACPIFTKIQGTCYRGKAKCCK	65.1	256.0
NA	DSHAKRHHGYKRKFHEKHHSHRGY	0.2	0.5
ATCC 19606	EKALEKLIAIQKAIKGMLNGWFTGVGFRRKR	8.0	28.5
M3237	EKALEKLIAIQKAIKGMLNGWFTGVGFRRKR	8.0	28.5
ATCC 17978	EKALEKLIAIQKAIKGMLNGWFTGVGFRRKR	8.0	28.5
CCARM 12036	FAHHIFRGIVHVGKTIHRLVTG	4.0	10.0

CCARM 12005	FAHHIFRGIVHVGKTIHRLVTG	4.0	10.0
CCARM 12035	FAHHIFRGIVHVGKTIHRLVTG	4.0	10.0
CCARM 12037	FAHHIFRGIVHVGKTIHRLVTG	4.0	10.0
ATCC 19606	FAKGIAGMAGKLF	200.0	262.1
NA	FALGAVTKRLPSLFCLITRKC	4.0	9.4
ATCC 19606	FASGIAGMAGKLF	200.0	253.9
ATCC 19606	FFFLRRIF	100.0	114.5
ATCC 19606	FFFLSRIF	100.0	107.6
ATCC 19606	FFGRLKSVWSAVKHGWKAAKSR	4.2	10.8
CCARM 12036	FFHHIARGIVHVGKTIHRLVTG	4.0	10.0
CCARM 12005	FFHHIARGIVHVGKTIHRLVTG	4.0	10.0
CCARM 12035	FFHHIARGIVHVGKTIHRLVTG	4.0	10.0
CCARM 12037	FFHHIARGIVHVGKTIHRLVTG	4.0	10.0
CCARM 12036	FFHHIFRGIKHVGKTIHRLVTG	2.0	5.2
CCARM 12005	FFHHIFRGIKHVGKTIHRLVTG	2.0	5.2
CCARM 12035	FFHHIFRGIKHVGKTIHRLVTG	2.0	5.2
CCARM 12037	FFHHIFRGIKHVGKTIHRLVTG	2.0	5.2
NA	FFHHIFRGIVHKGKTIHRLVTG	1.5	3.9
ATCC 19606	FFHHIFRGIVHKGKTIHRLVTG	3.0	7.8
ATCC 17978	FFHHIFRGIVHKGKTIHRLVTG	3.0	7.8
ATCC 19606	FFHHIFRGKVHVGKTIHRLVTG	1.5	3.9
ATCC 17978	FFHHIFRGKVHVGKTIHRLVTG	3.0	7.8
NA	FFHHIFRGKVHVGKTIHRLVTG	6.0	15.5
ATCC 19606	FFHHIFRPIVHVGKTIHRLVTG	5.9	15.4
ATCC 17978	FFHHIFRPIVHVGKTIHRLVTG	5.9	15.4
NA	FFHHIFRPIVHVGKTIHRLVTG	5.9	15.4
CCARM 12036	FFHHIKRGIKHVGKTIHRLVTG	4.0	10.3
CCARM 12005	FFHHIKRGIKHVGKTIHRLVTG	4.0	10.3
CCARM 12035	FFHHIKRGIKHVGKTIHRLVTG	4.0	10.3
CCARM 12037	FFHHIKRGIKHVGKTIHRLVTG	4.0	10.3
CCARM 12036	FFHHIKRGIVHVGKTIHRLVTG	4.0	10.2
CCARM 12005	FFHHIKRGIVHVGKTIHRLVTG	4.0	10.2
CCARM 12035	FFHHIKRGIVHVGKTIHRLVTG	4.0	10.2
CCARM 12037	FFHHIKRGIVHVGKTIHRLVTG	4.0	10.2
NA	FFPVIGRILNGIL	6.0	8.8
CCARM 12036	FKHHIFRGIKHVGKTIHRLVTG	2.0	5.2
CCARM 12005	FKHHIFRGIKHVGKTIHRLVTG	2.0	5.2
CCARM 12035	FKHHIFRGIKHVGKTIHRLVTG	2.0	5.2
CCARM 12037	FKHHIFRGIKHVGKTIHRLVTG	2.0	5.2

CCARM 12036	FKHHIFRGIVHVGKTIHRLVTG	2.0	5.1
CCARM 12005	FKHHIFRGIVHVGKTIHRLVTG	2.0	5.1
CCARM 12035	FKHHIFRGIVHVGKTIHRLVTG	2.0	5.1
CCARM 12037	FKHHIFRGIVHVGKTIHRLVTG	2.0	5.1
ATCC 19606	FLFSLIPSAIGGLISAFK	20.0	37.6
ATCC 15308	FLGGLIKIVPAMICAVRKKC	115.7	250.0
SR 201346	FLGGLIKIVPAMICAVTKKCHHHHHH	10.9	32.0
NA	FLGGLIKIVPAMICAVTKKCHHHHHH	21.9	64.0
ATCC 15308	FLGGLIKPVPAMICAVRKKC	116.6	250.0
ATCC 15308	FLGGLIKPWWPWRR	69.0	125.0
ATCC 15308	FLGGLIKRPPAMICAVRKKC	113.5	250.0
ATCC 15308	FLGGLIKRVPAMICAVRKKC	113.4	250.0
ATCC 15308	FLGGLIKWKWPWWPWRR	13.5	31.3
ATCC 15308	FLGGLIKWPWWPWRR	31.3	62.5
ATCC 15308	FLGGLIKWWPWRR	18.2	31.3
ATCC 19606	FLKGIKGMLGKLF	25.0	36.3
ATCC 19606	FLKGIKGMLGKLL	25.0	35.4
ATCC 19606	FLKGIVGKLGKLF	25.0	35.5
ATCC 19606	FLKGIVGMLGKLF	3.0	4.3
ATCC 19606	FLKGIVGMLGKLL	6.0	8.3
ATCC 19606	FLKGIVGMLGKLW	6.0	8.8
NA	FLPAALAGIGGILGKLF	15.8	26.2
ATCC 19606	FLPAALAGIGGILGKLF	25.0	41.5
NA	FLPLIGRVLSGIL	24.0	33.5
ATCC 19606	FLPWFSKFLGRIL	12.0	19.5
ATCC 19606	FLSGIVGMLGKLF	6.0	8.3
ATCC 19606	FLSLIPHIVSGVASIAKHF	6.3	12.7
ATCC 19606	FLSLIPHIVSGVASLAIHF	200.0	404.3
ATCC 19606	FLSLIPHIVSGVASLAKHF	6.3	12.7
ATCC 19606	FLSMIPHIVSGVAALAKHL	6.3	12.5
ATCC 19606	FSFLSRIF	100.0	101.6
NA	FSTKTRNWFSEHFKKVKEKLKDTFA	80.6	250.0
ATCC 19606	FVPWFSKFLGRIL	12.5	20.1
ATCC 19606	FVPWFSKFLKRIL	3.1	5.3
ATCC 19606	FVPWFSKFLPRIL	50.0	82.5
ATCC 19606	FVPWFSKFLWRIL	3.1	5.4
ATCC 19606	FVQWFSKFLGKIL	6.0	9.7
ATCC 19606	FVQWFSKFLGRIL	6.0	9.8
ATCC 19606	FVQWFSKFLLRIL	48.0	81.5

ATCC 19606	FVQWFSRFLGRIL	6.0	10.0
ATCC 19606	FVRWFSKFLGRIL	6.0	10.0
ATCC 19606	FVRWFSRFLGRIL	6.0	10.2
NA	FWGKLWEGVKNAI	38.0	58.8
ATCC 19606	GCKKYRRFRWKFKGKFWFW	24.1	64.0
ATCC 19606	GCKKYRRFRWKFKGKFWFWGG	23.1	64.0
ATCC 19606	GCRALCYKQRCVTYCRGA	8.0	16.4
ATCC 19606	GCRRFKKFKKWRYRGRFWFWCFG	20.3	64.0
ATCC 19606	GCRRLCYKQRCVTYCRGPPR	1.0	2.4
ATCC 19606	GCRRLCYKQRCVTYCRGR	1.0	2.2
ATCC 19606	GCRRWKKFRWRYRGKFWFWCG	22.0	64.0
ATCC 19606	GFCWYVCVYRNGVRVCYRRCN	0.2	0.5
NM8	GFGSLLGKALRLGANVL	3.0	5.1
NA	GFGSLLGKALRLGANVL	3.1	5.2
ATCC 19606	GFGSLLGKALRLGANVL	4.7	8.0
NA	GFLGPLLKLGLKGVAKVLPHLIPSRQQ	12.5	36.0
NM8	GFLGPLLKLGLKGVAKVLPHLIPSRQQ	12.5	36.0
NM8	GFLGSLLKTGLKVGSNLL	6.0	10.9
NM8	GFSSIFRGVAKFASKGLGKDLAKLGVDLVA	50.0	152.6
NM8	GFSSIFRGVAKFASKGLGKDLAKLGVDLVACKISKQC	6.0	23.1
NM8	GFSSIFRGVAKFASKGLGKDLAKLGVDLVASKISKQS	12.5	47.6
NM35	GFSSIFRGVAKFASKGLGKKLAKLGVKLVACKISKQC	1.5	5.8
NM8	GFSSIFRGVAKFASKGLGKKLAKLGVKLVACKISKQC	3.0	11.6
NA	GFWGKLWEGVKNAI	7.0	11.2
NA	GFWSSALEGLKKFAKGGLEALTNPK	12.5	33.1
NM8	GFWSSALEGLKKFAKGGLEALTNPK	12.5	33.1
AB3	GGLKKLGKKLEGAGKRVFKASEKALPVVVGIKAIGK	0.3	1.0
AB1	GGLKKLGKKLEGAGKRVFKASEKALPVVVGIKAIGK	0.5	2.0
ATCC 17978	GGLKKLGKKLEGAGKRVFKASEKALPVVVGIKAIGK	0.5	2.0
ATCC 17978	GGLKKLGKKLEGAGKRVFNAAEKALPVVAGAKALRK	0.5	2.0
NM35	GIFPIFAKLLGKVIKVASSLISKGRTE	10.0	28.7
NA	GIFPIFAKLLGKVIKVASSLISKGRTE	20.0	57.5
NM8	GIFPIFAKLLGKVIKVASSLISKGRTE	20.0	57.5
NA	GIGAVLKVLTTGLPALISWIKRKRQQ	4.2	12.1
NA	GIGDPVTCLKSGAICHPVFCPRRYKQIGTCGLPGTKCCKKP	29.5	128.0
ATCC 19606	GIGK	100.0	37.3
ATCC 19606	GIGKFLHSAGKFGKAFVGEIMKS	26.6	64.0
ATCC 19606	GIGKFLHSAKKFGKAFVGEIMNS	20.0	49.3
NA	GILKTIKSIASKLKRKAK	1.6	3.1

NA	GILKTIKSIASKVANTVQKLKRKAKNAV	3.1	9.3
NA	GILKTIKSIASKVANTVQKLKRKAKNAVA	3.1	9.5
NA	GILNTIKSIASKLKRKAK	1.6	3.1
NA	GIWDTIKSMGKVFAGAILQNL	12.5	28.3
NA	GIWDTIKSMGKVFAGLILQNL	25.0	57.6
NM109	GIWKTIKSMGKVFAGAIKQNL	3.0	6.9
NM8	GIWKTIKSMGKVFAGAIKQNL	3.0	6.9
NM75	GIWKTIKSMGKVFAGAIKQNL	3.0	6.9
NM35	GIWKTIKSMGKVFAGAIKQNL	3.0	6.9
NM124	GIWKTIKSMGKVFAGAIKQNL	3.0	6.9
NA	GIWKTIKSMGKVFAGAIKQNL	6.3	14.3
NA	GIWKTIKSMGKVFAGAILQNL	12.5	28.4
NM75	GIWKTIKSMGKVFAGKIKQNL	3.0	7.0
NM109	GIWKTIKSMGKVFAGKIKQNL	6.0	14.1
NM8	GIWKTIKSMGKVFAGKIKQNL	6.0	14.1
NM35	GIWKTIKSMGKVFAGKIKQNL	6.0	14.1
NM124	GIWKTIKSMGKVFAGKIKQNL	6.0	14.1
NA	GIWKTIKSMGKVFAGKIKQNL	6.3	14.7
NM109	GIWKTIKSMGKVFAGKILQNL	1.5	3.5
NM124	GIWKTIKSMGKVFAGKILQNL	1.5	3.5
NM8	GIWKTIKSMGKVFAGKILQNL	3.0	7.0
NM75	GIWKTIKSMGKVFAGKILQNL	3.0	7.0
NM35	GIWKTIKSMGKVFAGKILQNL	3.0	7.0
NA	GIWKTIKSMGKVFAGKILQNL	6.9	16.0
NA	GIWSSIKNLASKAWNSDIGQSLRNKAAGAINKFVADKIGVTP SQAAS	5.0	24.4
ATCC 19606	GKKYRRFRWKFKGKWFWFG	6.1	16.0
ATCC 19606	GKKYRRFRWKFRKGRFWFWG	5.7	16.0
ATCC 19606	GKKYRRFWKFKGKWFWFWG	6.1	16.0
ATCC 17978	GKLTKDKLKRGAKKALNVASKVAPIVAAGASIAR	0.9	3.0
NA	GLASTIGSLLGKFAKGGAQAFLQPK	25.0	61.5
NM8	GLASTIGSLLGKFAKGGAQAFLQPK	25.0	61.5
ATCC 15308	GLFDIWAWWRWRR	16.9	31.3
ATCC 15308	GLFDIWKKLRWRR	17.6	31.3
ATCC 15308	GLFDIWKKWRWRR	16.9	31.3
ATCC 15308	GLFDIWKWWRWRR	8.2	15.6
ATCC 15308	GLFDKWAWWRWRR	33.5	62.5
NA	GLFKKLRRKIKKGFKKIFKRL	3.8	10.0
ATCC 9955	GLFKKLRRKIKKGFKKIFKRL	15.9	42.0

ATCC 9955	GLFKKLRRKIKKGFKKIFKRLPPIGVGVSIPLAGKR	1.3	5.2
NA	GLFKKLRRKIKKGFKKIFKRLPPIGVGVSIPLAGKR	1.3	5.2
NM8	GLGKDLAKLGVDLVACKISKQC	200.0	452.0
NM35	GLGKDLAKLGVDLVACKISKQC	200.0	452.0
NA	GLGSLLGKAFKIGLKTVGKMMGGAPREQ	4.0	11.4
NA	GLGSVLGKALKIGANLL	4.0	6.5
NM8	GLGSVLGKALKIGANLL	6.0	9.7
NM8	GLKEIFKAGLGSLVKGIAAHVAS	5.0	11.3
NM35	GLKEIFKAGLGSLVKGIAAHVAS	10.0	22.7
NM8	GLKKIFKAGLGSLKKGIAAHVAS	20.0	45.9
NM35	GLKKIFKAGLGSLKKGIAAHVAS	20.0	45.9
NM75	GLKKIFKAGLGSLVKGIAAHVAS	2.5	5.7
NM35	GLKKIFKAGLGSLVKGIAAHVAS	20.0	45.3
NM8	GLKKIFKAGLGSLVKGIAAHVAS	40.0	90.6
NM75	GLKKIFKAGLGSLVKGIKAHVAS	5.0	11.6
NM8	GLKKIFKAGLGSLVKGIKAHVAS	10.0	23.2
NM124	GLKKIFKAGLGSLVKGIKAHVAS	20.0	46.5
NM75	GLKKIFKKGLGSLVKGIAAHVAS	2.5	5.8
NM8	GLKKIFKKGLGSLVKGIAAHVAS	5.0	11.6
NM35	GLKKIFKKGLGSLVKGIAAHVAS	10.0	23.2
NA	GLLKPLLKIAAKVGSNLL	1.6	3.0
KCTC 2508	GLNALKKVFQGIHEAIKKINNHVQ	2.0	5.4
CCARM 12036	GLNALKKVFQGIHEAIKKINNHVQ	4.0	10.8
CCARM 12035	GLNALKKVFQGIHEAIKKINNHVQ	4.0	10.8
CCARM 12036	GLNALKKVFQGIHKAIKKINNHVQ	2.0	5.4
CCARM 12035	GLNALKKVFQGIHKAIKKINNHVQ	2.0	5.4
KCTC 2508	GLNALKKVFQGIHKAIKKINNHVQ	2.0	5.4
CCARM 12036	GLNALKKVFQPIHEAIKKINNHVQ	16.0	43.8
CCARM 12035	GLNALKKVFQPIHEAIKKINNHVQ	16.0	43.8
KCTC 2508	GLNALKKVFQPIHEAIKKINNHVQ	16.0	43.8
CCARM 12036	GLNALKKVFQPIHEAIKLINNHVQ	8.0	21.8
CCARM 12035	GLNALKKVFQPIHEAIKLINNHVQ	8.0	21.8
KCTC 2508	GLNALKKVFQPIHEAIKLINNHVQ	8.0	21.8
CCARM 12036	GLNALKKVFQPIHKAIKKINNHVQ	8.0	21.9
KCTC 2508	GLNALKKVFQPIHKAIKKINNHVQ	8.0	21.9
CCARM 12035	GLNALKKVFQPIHKAIKKINNHVQ	16.0	43.8
NA	GLVGTLLGHIGKAILG	62.5	94.9
NM124	GLVGTLLGHIGKAILG	250.0	379.7
NM75	GLVGTLLGHIGKAILG	250.0	379.7

NM8	GLVGTLLGHIGKAILG	250.0	379.7
NM35	GLVGTLLGHIGKAILG	250.0	379.7
NM109	GLVGTLLGHIGKAILG	250.0	379.7
NA	GLVGTLLGHIGKAILS	62.5	96.8
NM75	GLVGTLLGHIGKAILS	62.5	96.8
NM124	GLVGTLLGHIGKAILS	125.0	193.6
NM8	GLVGTLLGHIGKAILS	125.0	193.6
NM35	GLVGTLLGHIGKAILS	125.0	193.6
NM109	GLVGTLLGHIGKAILS	125.0	193.6
NA	GMASKAGSVLGKVAKVALKAAL	4.0	8.3
NM8	GMATKAGTALGKVAKAVIGAAL	25.0	50.0
ATCC 19606	GRKKRRQRRRGGWMWVTNLRTD	2.8	8.0
NA	GRKKRRQRRRGGWMWVTNLRTD	5.7	16.0
NA	GRLRNLIEKAGQNIRGKIQGIGRRIKDILKNLQPRPQV	1.1	4.7
ATCC 19606	GRRYKKFRWKFKGRWFWFG	6.1	16.0
Q13	GTAWRWHYRARS	82.8	128.0
Q12	GTAWRWHYRARS	82.8	128.0
NM8	GVIKSVLKGVAKTVALGML	6.0	11.3
NM35	GVIKSVLKGVAKTVALGML	6.0	11.3
NM109	GVIKSVLKGVAKTVALGML	12.5	23.6
NM75	GVIKSVLKGVAKTVALGML	12.5	23.6
NM124	GVIKSVLKGVAKTVALGML	12.5	23.6
NA	GWANTLKNVAGGLCKITGAA	19.3	37.5
NA	GWFKKAWRKVKNAGRRVLKGVGIHYGVGLI	2.4	8.0
Q13	HLRRINKLLTRIGLYRHAFG	3.3	8.0
NA	HLRRINKLLTRIGLYRHAFG	26.3	64.0
NA	IASKVANTVQKLKRKAKNAV	50.0	108.4
NA	IASKVANTVQKLKRKAKNAVA	6.3	14.0
ATCC 19606	IDWKKVDWKKVSKKTCKVMLKACKFLG	0.0	0.1
NM35	IKIPSFFRNILKKVGKEAVSLIAGALKQS	5.0	15.8
NM8	IKIPSFFRNILKKVGKEAVSLIAGALKQS	10.0	31.6
NA	IKLSKETKDNLKKVLKGAIKGAIAVAKMV	6.3	19.3
NA	IKLSKETKKNLKKVLKGAIKGAIAVAKMV	3.1	9.6
NA	IKLSKKTKDNLKKVLKGAIKGAIAVAKMV	3.1	9.6
NA	IKLSPETKDNLKKVLKGAIKGAIAVAKMV	6.3	19.2
NA	IKLSPETKKNLKKVLKGAIKGAIAVAKMV	3.1	9.5
NA	IKLSPKTKDNLKKVLKGAIKGAIAVAKMV	3.1	9.5
NA	IKLSPKTKKNLKKVLKGAIKGAIAVAKMV	3.1	9.5
NA	IKSIASKVANTVQKLKRKAKNAV	6.3	15.6

NA	IKSIASKVANTVQKLKRKAKNAVA	3.1	8.0
NM8	ILGAILPLVSGLLSNKL	128.0	220.3
NM8	ILGKLLKTAAGLLSNL	64.0	104.0
NM8	ILGKLLKTAAKLLSNL	4.0	6.8
NM8	ILGKLLSTAAGLLKNL	32.0	52.0
NM8	ILGKLLSTAAGLLSKL	128.0	204.5
NM8	ILGKLLSTAAGLLSNL	64.0	101.4
NM8	ILGKLLSTAAKLLSNL	8.0	13.2
NA	ILSAIWSGIKSLF	10.0	14.3
NA	INLKAIAALAKKLF	3.7	5.7
ATCC 19606	ISKRILTGKK	223.9	256.0
ATCC 15308	ISLNPPRSTIAMRAINNYRWRSKNQNTFLR	3.5	12.7
NA	IWSAIWSGIKGLL	14.0	20.2
ATCC 19606	KAAAKWAAKAAK	100.0	121.4
ATCC 19606	KAK	100.0	34.5
ATCC 19606	KCRRLCYRQRCVTYCRGR	1.0	2.3
ATCC 19606	KCRRYCYRQRCVTYCRGR	1.0	2.4
ATCC 15308	KESRAKKFQRQHMDSDSSPSSSSTYSNQMMRRRNMTQGRSK PVNTFVH	9.0	50.7
NM124	KFASKGLGKDLAKLGVDLVACKISKQC	100.0	282.1
NM8	KFASKGLGKDLAKLGVDLVACKISKQC	200.0	564.3
NA	KFFKRLLKSVRRAVKKFRKKPRLIGLSTLL	55.1	200.0
ATCC 19606	KFHEKHHSHRGY	20.5	32.0
CCARM 12036	KFHHIFRGIKHVGKTIHRLVTG	2.0	5.2
CCARM 12005	KFHHIFRGIKHVGKTIHRLVTG	2.0	5.2
CCARM 12035	KFHHIFRGIKHVGKTIHRLVTG	2.0	5.2
CCARM 12037	KFHHIFRGIKHVGKTIHRLVTG	2.0	5.2
CCARM 12036	KFHHIFRGIVHVGKTIHRLVTG	2.0	5.1
CCARM 12005	KFHHIFRGIVHVGKTIHRLVTG	2.0	5.1
CCARM 12035	KFHHIFRGIVHVGKTIHRLVTG	2.0	5.1
CCARM 12037	KFHHIFRGIVHVGKTIHRLVTG	2.0	5.1
Q12	KFVRLKIYCRDKNKGRGISF	26.3	64.0
Q13	KFVRLKIYCRDKNKGRGISF	52.7	128.0
ATCC 19606	KFWKLLKKALRLWAKVL	3.7	8.0
NA	KFWKLLKKALRLWAKVL	7.5	16.0
ATCC 19606	KFWKLLKKALRLWKKVL	3.6	8.0
NA	KFWKLLKKALRLWKKVL	3.6	8.0
ATCC 19606	KFWSLLKKALRLWANVL	3.8	8.0
NA	KFWSLLKKALRLWANVL	3.8	8.0

ATCC 19606	KGGGKWGGKGGK	25.0	27.9
ATCC 19606	KGGK	12.5	4.9
ATCC 19606	KGIVGMLGKLF	50.0	58.1
NM8	KGLGKDLAKLGVDLVACKISKQC	200.0	477.6
ATCC 19606	KIAKVALKALKIAKGALKAL	1.5	3.1
NA	KIAKVALKALKIAKGALKAL	1.5	3.1
ATCC 19606	KIAKVALKALKIAKVALKAL	1.5	3.1
NA	KIAKVALKALKIAKVALKAL	1.5	3.1
NA	KIKKGFKKIFKRLPPIGVGVSIPLAGKR	3.3	10.0
ATCC 9955	KIKKGFKKIFKRLPPIGVGVSIPLAGKR	9.1	28.0
ATCC 19606	KILGVSKKIMRRISKDILTGKK	6.4	16.0
ATCC 19606	KILRGVSKKIMRRILTGKK	0.9	2.0
ATCC 19606	KILRGVSKKIMRRISKDILTGKK	3.0	8.0
ATCC 19606	KILRGVSKKIMRTFLRR	3.8	8.0
ATCC 19606	KILRGVSKRILTGKK	75.4	128.0
ATCC 19606	KISKKIMRTFLRR	152.6	256.0
ATCC 19606	KISKKIMRTFLRRILTGKK	1.7	4.0
ATCC 19606	KISKKIMRTFLRRISKDILTGKK	1.4	4.0
ATCC 19606	KK	75.0	20.6
NA	KKCGFFCKLKNKLKSTGSRSNIAAGTHGGTFRV	56.4	200.0
NA	KKCKFFCKVKKKIKSIGFQIPIVSIPFK	60.9	200.0
ATCC 19606	KKEK	37.0	19.7
ATCC 19606	KKIMRTFLRR	94.9	128.0
ATCC 19606	KKIMRTFLRRISKDILTGKK	3.3	8.0
ATCC 19606	KKIMRTFLRRISKKILTGKK	3.3	8.0
ATCC 19606	KKKK	12.5	6.6
ATCC 19606	KKKLKKLKKKLK	50.0	75.6
ATCC 19606	KKKLLLLLLLLLKKK	50.0	90.3
ATCC 19606	KKLLKKLKKLLK	19.0	28.1
ATCC 19606	KKLLKLLLKLLK	4.5	6.5
NA	KKRLKKIFKKPMVIGVTIPF	1.7	4.0
NA	KKWRKLLKKLKKLL	0.5	1.0
NA	KKWRKLLKWLAKK	1.3	2.3
NA	KKWRWWLKALAKK	2.3	4.0
NA	KKWRWWLKALAKKLL	0.2	0.4
ATCC 19606	KLAKLAKKLAKLAK	196.9	300.0
ATCC 19606	KLK	100.0	38.8
ATCC 19606	KLKLLKLLKLLKLLK	15.0	27.1
ATCC 899	KLKSLLKTLSKAKKKKLKTLLKALSK	0.3	0.9

ATCC 821	KLKSLLKTLSKAKKKKLKTLLKALSK	0.3	0.9
ATCC 985	KLKSLLKTLSKAKKKKLKTLLKALSK	0.3	0.9
ATCC 1012	KLKSLLKTLSKAKKKKLKTLLKALSK	0.3	0.9
ATCC 884	KLKSLLKTLSKAKKKKLKTLLKALSK	0.3	0.9
ATCC 689	KLKSLLKTLSKAKKKKLKTLLKALSK	0.3	0.9
ATCC 649	KLKSLLKTLSKAKKKKLKTLLKALSK	0.3	0.9
ATCC 19606	KLKSLLKTLSKAKKKKLKTLLKALSK	0.7	2.1
ATCC 17978	KLKSLLKTLSKAKKKKLKTLLKALSK	0.7	2.1
ATCC 964	KLKSLLKTLSKAKKKKLKTLLKALSK	0.7	2.1
ATCC 759	KLKSLLKTLSKAKKKKLKTLLKALSK	0.7	2.1
ATCC 821	KLKSLLKTLSKAKKKLLKTALKALSK	0.3	0.9
ATCC 985	KLKSLLKTLSKAKKKLLKTALKALSK	0.3	0.9
ATCC 759	KLKSLLKTLSKAKKKLLKTALKALSK	0.3	0.9
ATCC 649	KLKSLLKTLSKAKKKLLKTALKALSK	0.3	0.9
ATCC 19606	KLKSLLKTLSKAKKKLLKTALKALSK	0.7	2.0
ATCC 17978	KLKSLLKTLSKAKKKLLKTALKALSK	0.7	2.0
ATCC 964	KLKSLLKTLSKAKKKLLKTALKALSK	0.7	2.0
ATCC 899	KLKSLLKTLSKAKKKLLKTALKALSK	0.7	2.0
ATCC 1012	KLKSLLKTLSKAKKKLLKTALKALSK	0.7	2.0
ATCC 884	KLKSLLKTLSKAKKKLLKTALKALSK	0.7	2.0
ATCC 689	KLKSLLKTLSKAKKKLLKTALKALSK	0.7	2.0
NA	KLLK	127.8	64.0
ATCC 19606	KLLLKWLLKLLK	100.0	150.9
ATCC 19606	KNLRRIIRKIIHIIKKYG	2.6	5.9
ATCC 15308	KPKDMTSSQWFKTQHVQPSPQASNSAMSIINKYTERSKDLNT FLH	10.0	52.1
ATCC 15308	KPKGMTSSQWFKIQHMQPSPQASNSAMKNINKHTKRSKDL NTFLH	1.2	6.3
ATCC 15308	KPPQFTWAQWFETQHINMTSQQSTNAMQVINNYQRRSKNQ NTFLL	10.0	54.6
NA	KRFKKFFKKLKNSVKKRAKKFFKKPRVIGVSIPF	3.9	16.0
NA	KRFKKFFKKLKNSVKKRVKKFFRKPRVIGVTFPF	0.3	1.1
NA	KRFKKFFKKVKKSV	71.2	128.0
NA	KRFKKFFKKVKKSVKKRLKKIFKKPMVIGVTIPF	0.3	1.0
NA	KRGFGKKLRKRLKKFRNSIKKRLKNFNVVIPIPLPG	1.9	8.1
ATCC 19606	KRGFGKKLRKRLKKFRNSIKKRLKNFNVVIPIPLPG	3.8	16.3
NA	KRIVQRIKDFLRNLVPRTES	6.5	16.0
ATCC 19606	KRIVQRIKDFLRNLVPRTES	25.9	64.0
NA	KRRGSVTTRYQFLMIHLLRPKKLFA	3.1	9.4
ATCC 17978	KRWWKWIRW	5.0	7.2

ATCC 17978	KRWWKWWRR	20.0	29.8
NA	KSKEKIGKEFKRIVQRIKDFLRNLVPRTES	2.2	8.0
ATCC 19606	KSKEKIGKEFKRIVQRIKDFLRNLVPRTES	4.4	16.0
NA	KTRNWFSEHFKKVKEKLKDTFA	90.3	250.0
NA	KVANTVQKLKRKAKNAVA	50.0	98.4
ATCC 19606	KVVVKWVVKVVK	100.0	141.1
NA	KWCFRVCYRGICYRKCR	7.1	16.0
ATCC 19606	KWCFRVCYRGICYRRCR	0.1	0.1
ATCC 19606	KWKIFKKIEKVGRNIRNGIIKAGPAVAVLGEAKAL	33.5	128.0
NA	KWKLFKKIGIGAVLKVLTTG	3.1	6.9
KCTC 2508	KWKLFKKIGIGAVLKVLTTG	3.1	6.9
NA	KWKLFKKIGIGKFLHSAKKF	3.1	7.5
KCTC 2508	KWKLFKKIGIGKFLHSAKKF	12.5	30.1
NA	KWKLFKKIPKFLHLAKKF	5.4	12.5
NA	KWKSFIKKLTKKFLHSAKKF	0.6	1.6
NA	KWKSFIKKLTSKFLHSAKKF	1.3	3.1
ATCC 19606	KWKSFLKTFKSAVKTVLHTALKAISS	0.7	2.0
ATCC 17978	KWKSFLKTFKSAVKTVLHTALKAISS	0.7	2.0
ATCC 899	KWKSFLKTFKSAVKTVLHTALKAISS	0.7	2.0
ATCC 821	KWKSFLKTFKSAVKTVLHTALKAISS	0.7	2.0
ATCC 985	KWKSFLKTFKSAVKTVLHTALKAISS	0.7	2.0
ATCC 1012	KWKSFLKTFKSAVKTVLHTALKAISS	0.7	2.0
ATCC 759	KWKSFLKTFKSAVKTVLHTALKAISS	0.7	2.0
ATCC 884	KWKSFLKTFKSAVKTVLHTALKAISS	0.7	2.0
ATCC 689	KWKSFLKTFKSAVKTVLHTALKAISS	0.7	2.0
ATCC 649	KWKSFLKTFKSAVKTVLHTALKAISS	0.7	2.0
ATCC 964	KWKSFLKTFKSAVKTVLHTALKAISS	1.3	3.8
ATCC 17978	KWKSFLKTFSKAKKKALKTLLKAISK	0.7	2.1
ATCC 964	KWKSFLKTFSKAKKKALKTLLKAISK	0.7	2.1
ATCC 899	KWKSFLKTFSKAKKKALKTLLKAISK	0.7	2.1
ATCC 821	KWKSFLKTFSKAKKKALKTLLKAISK	0.7	2.1
ATCC 985	KWKSFLKTFSKAKKKALKTLLKAISK	0.7	2.1
ATCC 1012	KWKSFLKTFSKAKKKALKTLLKAISK	0.7	2.1
ATCC 759	KWKSFLKTFSKAKKKALKTLLKAISK	0.7	2.1
ATCC 884	KWKSFLKTFSKAKKKALKTLLKAISK	0.7	2.1
ATCC 19606	KWKSFLKTFSKAKKKALKTLLKAISK	1.3	3.9
ATCC 689	KWKSFLKTFSKAKKKALKTLLKAISK	1.3	3.9
ATCC 649	KWKSFLKTFSKAKKKALKTLLKAISK	1.3	3.9
ATCC 19606	KWKSFLKTFSKAKKKKLKTLLKAISK	0.6	1.8
ATCC 899	KWKSFLKTFSKAKKKKLKTLLKAISK	0.6	1.8
ATCC 985	KWKSFLKTFSKAKKKKLKTLLKAISK	0.6	1.8
ATCC 1012	KWKSFLKTFSKAKKKKLKTLLKAISK	0.6	1.8
ATCC 759	KWKSFLKTFSKAKKKKLKTLLKAISK	0.6	1.8
ATCC 884	KWKSFLKTFSKAKKKKLKTLLKAISK	0.6	1.8
ATCC 649	KWKSFLKTFSKAKKKKLKTLLKAISK	0.6	1.8
ATCC 17978	KWKSFLKTFSKAKKKKLKTLLKAISK	1.2	3.7
ATCC 964	KWKSFLKTFSKAKKKKLKTLLKAISK	1.2	3.7
ATCC 821	KWKSFLKTFSKAKKKKLKTLLKAISK	1.2	3.7
ATCC 689	KWKSFLKTFSKAKKKKLKTLLKAISK	1.2	3.7
ATCC 899	KWKSFLKTFSKAKKKVLKTALKAISK	0.3	0.9

ATCC 759	KWKSFLKTFSKAKKKVLKTALKAISK	0.3	0.9
ATCC 884	KWKSFLKTFSKAKKKVLKTALKAISK	0.3	0.9
ATCC 649	KWKSFLKTFSKAKKKVLKTALKAISK	0.3	0.9
ATCC 19606	KWKSFLKTFSKAKKKVLKTALKAISK	0.7	2.1
ATCC 17978	KWKSFLKTFSKAKKKVLKTALKAISK	0.7	2.1
ATCC 821	KWKSFLKTFSKAKKKVLKTALKAISK	0.7	2.1
ATCC 985	KWKSFLKTFSKAKKKVLKTALKAISK	0.7	2.1
ATCC 689	KWKSFLKTFSKAKKKVLKTALKAISK	0.7	2.1
ATCC 964	KWKSFLKTFSKAKKKVLKTALKAISK	1.3	3.9
ATCC 1012	KWKSFLKTFSKAKKKVLKTALKAISK	1.3	3.9
ATCC 19606	KWLKKWLKWLKK	4.7	8.0
NA	KWWKWWKKWWKK	3.3	6.3
ATCC 19606	LFWKLLLKALRLWAKVL	3.8	8.0
NA	LFWKLLLKALRLWAKVL	3.8	8.0
NA	LGAWLAGKVAGTVATYAWNRYV	25.0	59.2
ATCC 19606	LIRGLFKSFWQVF	6.0	9.8
NA	LKAAAAAAKLAAKAAKAALKAAAAAAKL	3.2	8.1
NA	LKFLKFG	128.0	109.1
NA	LKLKAIAALAKKKW	5.1	8.0
NA	LKLKSIVSWAKKVL	1.2	2.0
NA	LKLLKKLLKKLLKLL	3.1	5.6
ATCC 19606	LKLLKKLLKKLLKLL	13.0	23.5
NA	LKLSPKTKDTLKKVLKGAIKGAIAIASMA	0.6	1.8
NA	LKWLKWG	128.0	119.1
NA	LLKKALRLWKKVL	19.9	32.0
ATCC 19606	LLKKALRLWKKVL	79.5	128.0
NA	LLKKLLKKC	18.4	20.0
ATCC 19606	LLKKLLKLLLKLLKK	11.0	19.9
ATCC 19606	LLKLLKKLLKKLLKL	10.0	18.1
ATCC 19606	LLLLLKKKKKKLLLL	50.0	90.3
ATCC 15308	LLPWKWPWWKWRR	129.0	250.0
ATCC 19606	LLQWLSKLLGRLL	12.0	18.6
ATCC 19606	LLQWLSKLLGRWL	6.0	9.8
ATCC 19606	LLWKALRLWWKVL	9.3	16.0
NA	LLWKALRLWWKVL	9.3	16.0
Q13	LPRRNRWSKIWKKVVTVFS	1.7	4.0
NA	LPRRNRWSKIWKKVVTVFS	9.4	22.6
NA	LRDLVCYCRTRGCKRRERMNGTCRKGHLMYTLCCR	30.1	128.0
NA	LRKLRKRLLLRKLRKRLL	13.5	32.0
NA	LRKLRKRLVRLASHLRKLRKRLL	2.1	6.0
NA	LRWLRWG	128.0	126.2
ATCC 19606	LRWTPTPSYPRYPTRSRGSRWSR	8.0	22.9
NA	LVQRGRFGRFLKKVRRFIPKVIIAAQIGSRFG	1.3	4.8
ATCC 17978	NPEKALEKLIAIQKAIKGMLNGWFTGVGFRRKR	4.0	15.1
M6337	NPEKALEKLIAIQKAIKGMLNGWFTGVGFRRKR	4.0	15.1
ATCC 19606	NPEKALEKLIAIQKAIKGMLNGWFTGVGFRRKR	8.0	30.2
M105656	NPEKALEKLIAIQKAIKGMLNGWFTGVGFRRKR	8.0	30.2
M2925	NPEKALEKLIAIQKAIKGMLNGWFTGVGFRRKR	8.0	30.2
M3237	NPEKALEKLIAIQKAIKGMLNGWFTGVGFRRKR	16.0	60.3
ATCC 19606	NPEKALEPLIAIQIAIKGMLNGWFTGVGFRRKR	64.0	238.4
M3237	NPEKALEPLIAIQIAIKGMLNGWFTGVGFRRKR	64.0	238.4

ATCC 17978	NPEKALEPLIAIQIAIKGMLNGWFTGVGFRRKR	64.0	238.4
Q12	NRFTARFRRTPWRLCLQFRQ	3.0	8.0
Q13	NRFTARFRRTPWRLCLQFRQ	12.1	32.0
NA	NRFTARFRRTPWRLCLQFRQ	17.1	45.3
NA	PPPVIKFNRPFLMWIVERDTRSILFMGKIVNPKAP	60.8	250.0
ICU 63169	PRPGPRP	165.0	128.0
ICU 63169	PRPLPRP	153.8	128.0
ICU 63169	PRPRPRP	146.3	128.0
ICU 63169	PRPWPRP	141.4	128.0
ATCC 15308	QDGMYQRFLRQHVHPEETGGSDRYSNLMMQRRKMTLYHSK RFNTFIH	0.5	2.9
ATCC 15308	QDNSRYTHFLTQHYDAKPQGRDDRYSESIMRRRGLTSPSKDI NTFIH	10.0	56.4
AB1	QKKIRVRLSA	13.4	16.0
AB7	QKKIRVRLSA	26.7	32.0
Q12	QVRWWGRYWRRKWATCR	6.7	16.0
Q13	QVRWWGRYWRRKWATCR	26.7	64.0
NA	RGGLCYCRGRFCVCVGR	2.1	4.0
NM124	RGVAKFASKGLGKDLAKLGVDLVACKISKQC	50.0	160.2
NM8	RGVAKFASKGLGKDLAKLGVDLVACKISKQC	100.0	320.5
NM35	RGVAKFASKGLGKDLAKLGVDLVACKISKQC	200.0	641.0
NA	RIKRFWPVVIRTVVAGYNLYRAIKKK	0.7	2.4
ATCC 19606	RILRGVSRRIMRRILTGRR	3.4	8.0
ATCC 17978	RKWWRWIKW	5.0	7.2
ATCC 15308	RLPWRWPRRPWRR	130.3	250.0
AB5075	RLVRILVSKRPVAIKPYFRL	2.0	4.9
ATCC 15308	RPPQFTRAQWFAIQHISLN	10.0	23.1
ATCC 15308	RPPQFTRAQWFAIQHISLNPPRCTIAMRAINNYRWRCKNQN TFLRTTFANVVNVCGNQSIRCPHNRTLNNCHRSRFRVPLLHC DLINPGAQNISNCTYADRPGRRFYVVACDNRDPRDSPRYPVV PVHLDTTI	0.3	4.8
ATCC 15308	RPPQFTRAQWFAIQHISLNPPRSTIAMRAINNYRWRSKNQNT FL	0.3	1.6
ATCC 15308	RPPQFTRAQWFAIQHISLNPPRSTIAMRAINNYRWRSKNQNT FLR	0.6	3.3
NA	RRGLFKKLRRKIKKGFKKIFKRLPPVGVGVSIPLAGRR	1.1	4.7
NA	RRIRPRPPRLPRPRPRPLPFPRPGPRPIPRPLPFP	0.5	2.1
ATCC 15308	RRPWRWPRWPWRR	125.5	250.0
ATCC 15308	RRPWRWPWWPWRR	123.7	250.0
ATCC 19606	RRRRRFRRVIRRIRLPKYLTINTE	2.0	6.5
ATCC 19606	RRRRRYRYWRRGLTIQGRPKSLPLNTGD	2.0	7.1
NA	RRSKARGGSRGSKMGRKDSKGGSRGRPGSGSRPGGGSSIAGA SRGDRGGTRNA	4.7	24.4
AB1	RRWVRRVRRWVRRVVRVVRRWVRR	1.5	5.0
AB3	RRWVRRVRRWVRRVVRVVRRWVRR	1.8	6.0
AB2	RRWVRRVRRWVRRVVRVVRRWVRR	3.2	11.0
Q13	RSITRPVLVRRRWRVRPVF	52.2	128.0
Q12	RSITRPVLVRRRWRVRPVF	52.2	128.0
NA	RVRRFWPLVPVAINTVAAGINLYKAIRRK	2.8	9.5
NA	RWKIFKKIEKMGRNIRDGIVKAGPAIEVLGSAKAIGK	1.4	5.7
CCARM 12035	RWKIFKKIEKVGRNVRDGIIKAGPAVAVVGQAATVVK	1.0	4.0

CCARM 12037	RWKIFKKIEKVGRNVRDGIIKAGPAVAVVGQAATVVK	1.0	4.0
CCARM 12036	RWKIFKKIEKVGRNVRDGIIKAGPAVAVVGQAATVVK	1.0	4.0
CCARM 12005	RWKIFKKIEKVGRNVRDGIIKAGPAVAVVGQAATVVK	2.0	8.0
CCARM 12035	RWKIFKKIPKFLHSAKKF	2.0	4.6
CCARM 12037	RWKIFKKIPKFLHSAKKF	2.0	4.6
CCARM 12036	RWKIFKKIPKFLHSAKKF	2.0	4.6
CCARM 12005	RWKIFKKIPKFLHSAKKF	2.0	4.6
DSM 30007	RWRWRW	85.0	88.8
AB2	RWWRWWRRWWRR	1.4	3.0
AB1	RWWRWWRRWWRR	2.4	5.0
NA	RWWRWWRRWWRR	2.7	5.6
AB3	RWWRWWRRWWRR	5.3	11.0
NA	SAVGRHGRRFGLRKHRKH	50.0	107.8
CICC 22934	SAVGRHGRRFGLRKHRKH	100.0	215.6
CICC 22934	SAVGRHLRRFGLRKHRKH	100.0	221.2
CICC 22934	SAVGRHLRRFLLRKHRKH	100.0	226.8
CICC 22934	SAVLRHLRRFLLRKHRKH	100.0	232.4
Q12	SIKILKIYFIQGKRHWSF	14.1	32.0
Q13	SIKILKIYFIQGKRHWSF	28.3	64.0
CICC 22934	SKVGRHGRRFGHRAHRKL	100.0	215.6
CICC 22934	SKVGRHLRRFGHRAHRKL	100.0	221.2
CICC 22934	SKVGRHLRRFLHRAHRKL	100.0	226.8
CICC 22934	SKVLRHLRRFLHRAHRKL	100.0	232.4
CICC 22934	SKVWRHWRRFWHRAHRKK	7.8	20.0
NA	SKVWRHWRRFWHRAHRLH	25.0	63.8
ATCC 19606	SMATPHVAGAAALILSKHPTWTNAQVRDRLESTATYLGNSF YYGK	26.1	128.0
SRAC2	SMATPHVAGAAALILSKHPTWTNAQVRDRLESTATYLGNSF YYGK	26.1	128.0
ATCC 15308	TIAMRAINNYRWRSKNQNTFLR	1.1	3.0
Q12	TMSLRFWRWKVR	9.6	16.0
Q13	TMSLRFWRWKVR	19.2	32.0
NA	TMSLRFWRWKVR	76.8	128.0
NA	TRWLWLLRGGLKAAGWGIRAHLNRNQ	65.7	200.0
AB5075	TTSIRRRYQVSLIRRHRGKR	1.0	2.5
DSM 3008	TWLKKRRWKKVKPP	34.6	64.0
AB1	VAKGLIKGVKAKGELPAKGVFKGLKESIGKRAVLKG	8.7	32.0
ATCC 17978	VAKGLIKGVKAKGELPAKGVFKGLKESIGKRAVLKG	8.7	32.0
DSM 30008	VDKPPYLPRPRPPRRIYNR	6.7	16.0
ATCC 19606	VKGSWSKKFEVIA	1.6	2.3
NA	VKGSWSKKFEVIA	3.1	4.6
Q12	VLHTGYRKFLHRSKRFFHLR	24.6	64.0
Q13	VLHTGYRKFLHRSKRFFHLR	49.2	128.0
NA	VQLRIRVAVIRA	23.0	32.0
NA	VQLRIRVCVIRK	10.8	16.0
NA	VQLRIRVCVIRR	21.2	32.0
NA	VQWRIRIAVIRA	10.8	16.0
NA	VQWRIRVAVIRK	5.2	8.0
NA	VWLSALKFIGKHLAKHQLSKL	3.1	7.5
ATCC 19606	WFKKLLKKALRLWKKVL	3.6	8.0
NA	WFKKLLKKALRLWKKVL	7.3	16.0

ATCC 19606	WFWKLLWKALRLWWKVL	6.7	16.0
NA	WFWKLLWKALRLWWKVL	6.7	16.0
ATCC 19606	WGRRWRIRIPRLPRPWPPRPKWPRSATINTDQ	2.0	8.1
ATCC 19606	WKRRIKIWKKIR	256.0	438.1
NA	WKRRIKIWKKIR	256.0	438.1
ATCC 19606	WLRRIKAWLRR	16.0	24.9
NA	WLRRIKAWLRR	64.0	99.5
ATCC 19606	WLRRIKAWLRRIKA	2.0	3.7
NA	WLRRIKAWLRRIKA	2.0	3.7
NA	WLRRIKAWLRRKRK	4.0	7.9
ATCC 19606	WLRRIKAWLRRKRK	16.0	31.5
ATCC 15308	WPKRLTKAHWFEIQHIQPSPLQCNRAMSGINNYTQHCKHQ NTFLH	0.9	5.1
ATCC 15308	WPKRLTKAHWFEIQHIQPSPLQCNRAMSGINNYTQHCKHQ NTFLHDSFQNVAAVCDLLSIVCKNRRHNCHQSSKPVNMTD CRLTSGKYPQCRYSAAAQYKFFIVACDPPQKSDPPYKLVPVH LDSIL	0.6	9.1
ATCC 15308	WPKRLTKAHWFEIQHIQPSPLQSNRAMSGINNYTQHSKHQN TFLH	1.2	6.5
ATCC 19606	WPRFPKPRKPTYPGPTYPGPTWPRPTWRRSATIDTEH	32.0	141.8
ATCC 19606	WW	145.5	56.8
ATCC 19606	YSWPRMPRIPRLPRYPRYPRYPRWPRHPTIYA	1.0	4.2
ATCC 19606	YSWPRMPRIPRLPRYPRYPRYPRWPRWPRQPTIYA	4.0	18.5

Table S2: Dataset 1 containing unique antimicrobial peptides with activity against *A. baumannii*. By removing duplicate sequences from the Table S1 peptide list, we generated **Dataset 1 (**374 sequences), and this dataset was used for designing novel HRZN peptides using our "DFT+PA" method.

**Strain**	**Sequence**	**µM**	**µg/mL**
ATCC 19606	IDWKKVDWKKVSKKTCKVMLKACKFLG	0.0	0.1
ATCC 19606	KWCFRVCYRGICYRRCR	0.1	0.1
NA	KKWRWWLKALAKKLL	0.2	0.4
ATCC 19606	GFCWYVCVYRNGVRVCYRRCN	0.2	0.5
NA	DSHAKRHHGYKRKFHEKHHSHRGY	0.2	0.5
ATCC 17978	ALKSLLKTLSAAKKKKLATLLKALSK	0.2	0.6
ATCC 821	KLKSLLKTLSKAKKKLLKTALKALSK	0.3	0.9
ATCC 899	KLKSLLKTLSKAKKKKLKTLLKALSK	0.3	0.9
ATCC 899	KWKSFLKTFSKAKKKVLKTALKAISK	0.3	0.9
NA	CYCRIPACIAGERRYGTCIYQGRLWAFCC	0.3	1.0
NA	DCYCRIPACIAGERRYGTCIYQGRLWAFCC	0.3	1.0
AB3	GGLKKLGKKLEGAGKRVFKASEKALPVVVGIKAIGK	0.3	1.0
NA	KKWRKLLKKLKKLL	0.5	1.0
NA	KRFKKFFKKVKKSVKKRLKKIFKKPMVIGVTIPF	0.3	1.0
M89955	ALKSLLKTLSAAAKKALATLLKALSK	0.4	1.1
NA	KRFKKFFKKLKNSVKKRVKKFFRKPRVIGVTFPF	0.3	1.1
ATCC 19606	ALKSLLATLSKAKKKKLKTLLAALSK	0.4	1.1
ATCC 19606	ALWMTLKKKVLKAAAKALNAVLVGANA	0.4	1.1
ATCC 15308	RPPQFTRAQWFAIQHISLNPPRSTIAMRAINNYRWRSKNQNT FL	0.3	1.6
NA	KWKSFIKKLTKKFLHSAKKF	0.6	1.6

NA	LKLSPKTKDTLKKVLKGAIKGAIAIASMA	0.6	1.8
ATCC 19606	KWKSFLKTFSKAKKKKLKTLLKAISK	0.6	1.8
M89955	ALKSLLATLSKAAKKALKTLLAALSK	0.7	1.9
ATCC 19606	KILRGVSKKIMRRILTGKK	0.9	2.0
ATCC 17978	GGLKKLGKKLEGAGKRVFNAAEKALPVVAGAKALRK	0.5	2.0
NA	LKLKSIVSWAKKVL	1.2	2.0
ATCC 19606	KWKSFLKTFKSAVKTVLHTALKAISS	0.7	2.0
NA	RRIRPRPPRLPRPRPRPLPFPRPGPRPIPRPLPFP	0.5	2.1
ATCC 17978	KWKSFLKTFSKAKKKALKTLLKAISK	0.7	2.1
ATCC 19606	GCRRLCYKQRCVTYCRGR	1.0	2.2
NA	KKWRKLLKWLAKK	1.3	2.3
ATCC 19606	VKGSWSKKFEVIA	1.6	2.3
ATCC 19606	KCRRLCYRQRCVTYCRGR	1.0	2.3
NA	RIKRFWPVVIRTVVAGYNLYRAIKKK	0.7	2.4
ATCC 19606	KCRRYCYRQRCVTYCRGR	1.0	2.4
ATCC 19606	GCRRLCYKQRCVTYCRGPPR	1.0	2.4
AB5075	TTSIRRRYQVSLIRRHRGKR	1.0	2.5
ATCC 15308	QDGMYQRFLRQHVHPEETGGSDRYSNLMMQRRKMTLYHSK RFNTFIH	0.5	2.9
NA	GLLKPLLKIAAKVGSNLL	1.6	3.0
ATCC 17978	GKLTKDKLKRGAKKALNVASKVAPIVAAGASIAR	0.9	3.0
AB2	RWWRWWRRWWRR	1.4	3.0
ATCC 15308	TIAMRAINNYRWRSKNQNTFLR	1.1	3.0
NA	GILNTIKSIASKLKRKAK	1.6	3.1
ATCC 19606	KIAKVALKALKIAKGALKAL	1.5	3.1
NA	GILKTIKSIASKLKRKAK	1.6	3.1
NA	KWKSFIKKLTSKFLHSAKKF	1.3	3.1
ATCC 19606	KIAKVALKALKIAKVALKAL	1.5	3.1
ATCC 15308	RPPQFTRAQWFAIQHISLNPPRSTIAMRAINNYRWRSKNQNT FLR	0.6	3.3
NM109	GIWKTIKSMGKVFAGKILQNL	1.5	3.5
NA	ALASLLKTLSKAAKKALKTLLKALSA	1.4	3.7
ATCC 19606	WLRRIKAWLRRIKA	2.0	3.7
ATCC 19606	ALWKKLLKKLLKSAKKLG	1.9	3.9
ATCC 19606	FFHHIFRGKVHVGKTIHRLVTG	1.5	3.9
NA	RGGLCYCRGRFCVCVGR	2.1	4.0
ATCC 19606	KISKKIMRTFLRRILTGKK	1.7	4.0
ATCC 19606	KISKKIMRTFLRRISKDILTGKK	1.4	4.0
NA	KKRLKKIFKKPMVIGVTIPF	1.7	4.0
Q13	LPRRNRWSKIWKKVVTVFS	1.7	4.0
NA	KKWRWWLKALAKK	2.3	4.0
CCARM 12035	RWKIFKKIEKVGRNVRDGIIKAGPAVAVVGQAATVVK	1.0	4.0
ATCC 15308	AQWFAIQHISLNPPRSTIAMRAINNYRWRSKNQNTFLR	0.9	4.0
ATCC 19606	YSWPRMPRIPRLPRYPRYPRYPRWPRHPTIYA	1.0	4.2
ATCC 19606	FLKGIVGMLGKLF	3.0	4.3
CCARM 12035	RWKIFKKIPKFLHSAKKF	2.0	4.6
NA	RRGLFKKLRRKIKKGFKKIFKRLPPVGVGVSIPLAGRR	1.1	4.7
NA	GRLRNLIEKAGQNIRGKIQGIGRRIKDILKNLQPRPQV	1.1	4.7

ATCC 15308	RPPQFTRAQWFAIQHISLNPPRCTIAMRAINNYRWRCKNQN TFLRTTFANVVNVCGNQSIRCPHNRTLNNCHRSRFRVPLLHC DLINPGAQNISNCTYADRPGRRFYVVACDNRDPRDSPRYPVV PVHLDTTI	0.3	4.8
NA	LVQRGRFGRFLKKVRRFIPKVIIAAQIGSRFG	1.3	4.8
AB5075	RLVRILVSKRPVAIKPYFRL	2.0	4.9
ATCC 19606	KGGK	12.5	4.9
CCARM 12036	AFHHIFRGIVHVGKTIHRLVTG	2.0	5.0
AB1	RRWVRRVRRWVRRVVRVVRRWVRR	1.5	5.0
NM8	GFGSLLGKALRLGANVL	3.0	5.1
ATCC 15308	WPKRLTKAHWFEIQHIQPSPLQCNRAMSGINNYTQHCKHQ NTFLH	0.9	5.1
CCARM 12036	FKHHIFRGIVHVGKTIHRLVTG	2.0	5.1
CCARM 12005	KFHHIFRGIVHVGKTIHRLVTG	2.0	5.1
CCARM 12036	FKHHIFRGIKHVGKTIHRLVTG	2.0	5.2
CCARM 12036	KFHHIFRGIKHVGKTIHRLVTG	2.0	5.2
ATCC 9955	GLFKKLRRKIKKGFKKIFKRLPPIGVGVSIPLAGKR	1.3	5.2
CCARM 12036	FFHHIFRGIKHVGKTIHRLVTG	2.0	5.2
ATCC 19606	FVPWFSKFLKRIL	3.1	5.3
ATCC 15308	AQWFAIQHISLNPPRSTIAMRAINNYRWR	1.5	5.3
CCARM 12036	GLNALKKVFQGIHKAIKKINNHVQ	2.0	5.4
KCTC 2508	GLNALKKVFQGIHEAIKKINNHVQ	2.0	5.4
ATCC 19606	FVPWFSKFLWRIL	3.1	5.4
NA	LKLLKKLLKKLLKLL	3.1	5.6
NA	INLKAIAALAKKLF	3.7	5.7
NM75	GLKKIFKAGLGSLVKGIAAHVAS	2.5	5.7
NA	RWKIFKKIEKMGRNIRDGIVKAGPAIEVLGSAKAIGK	1.4	5.7
NM35	GFSSIFRGVAKFASKGLGKKLAKLGVKLVACKISKQC	1.5	5.8
ATCC 19606	KNLRRIIRKIIHIIKKYG	2.6	5.9
NA	LRKLRKRLVRLASHLRKLRKRLL	2.1	6.0
ATCC 15308	KPKGMTSSQWFKIQHMQPSPQASNSAMKNINKHTKRSKDL NTFLH	1.2	6.3
NA	KWWKWWKKWWKK	3.3	6.3
ATCC 19606	RRRRRFRRVIRRIRLPKYLTINTE	2.0	6.5
ATCC 15308	WPKRLTKAHWFEIQHIQPSPLQSNRAMSGINNYTQHSKHQN TFLH	1.2	6.5
NA	GLGSVLGKALKIGANLL	4.0	6.5
ATCC 19606	KKLLKLLLKLLK	4.5	6.5
ATCC 19606	KKKK	12.5	6.6
NM8	ILGKLLKTAAKLLSNL	4.0	6.8
NA	KWKLFKKIGIGAVLKVLTTG	3.1	6.9
NM109	GIWKTIKSMGKVFAGAIKQNL	3.0	6.9
NM75	GIWKTIKSMGKVFAGKIKQNL	3.0	7.0
ATCC 19606	RRRRRYRYWRRGLTIQGRPKSLPLNTGD	2.0	7.1
NA	AKKVFKRLGIGKFLHSAKKF	3.1	7.2
ATCC 17978	KRWWKWIRW	5.0	7.2
ATCC 17978	RKWWRWIKW	5.0	7.2
NA	VWLSALKFIGKHLAKHQLSKL	3.1	7.5
NA	AMVSS	15.2	7.5

NA	KWKLFKKIGIGKFLHSAKKF	3.1	7.5
ATCC 17978	ALKSLLKTLSKAAAAALKTLLKALSK	2.9	7.7
NA	WLRRIKAWLRRKRK	4.0	7.9
ATCC 19606	RILRGVSRRIMRRILTGRR	3.4	8.0
ATCC 19606	GRKKRRQRRRGGWMWVTNLRTD	2.8	8.0
ATCC 19606	KILRGVSKKIMRTFLRR	3.8	8.0
ATCC 19606	KILRGVSKKIMRRISKDILTGKK	3.0	8.0
ATCC 19606	KKIMRTFLRRISKDILTGKK	3.3	8.0
ATCC 19606	KKIMRTFLRRISKKILTGKK	3.3	8.0
Q12	NRFTARFRRTPWRLCLQFRQ	3.0	8.0
NA	KSKEKIGKEFKRIVQRIKDFLRNLVPRTES	2.2	8.0
Q13	HLRRINKLLTRIGLYRHAFG	3.3	8.0
NA	VQWRIRVAVIRK	5.2	8.0
NA	GWFKKAWRKVKNAGRRVLKGVGIHYGVGLI	2.4	8.0
ATCC 19606	AGYLLGKINLKALAALAKKIL	3.7	8.0
ATCC 19606	AAYLLAKINLKALAALAKKIL	3.6	8.0
NA	LKLKAIAALAKKKW	5.1	8.0
ATCC 19606	KFWSLLKKALRLWANVL	3.8	8.0
ATCC 19606	KFWKLLKKALRLWAKVL	3.7	8.0
ATCC 19606	KFWKLLKKALRLWKKVL	3.6	8.0
ATCC 19606	WFKKLLKKALRLWKKVL	3.6	8.0
ATCC 19606	LFWKLLLKALRLWAKVL	3.8	8.0
ATCC 19606	KWLKKWLKWLKK	4.7	8.0
NA	IKSIASKVANTVQKLKRKAKNAVA	3.1	8.0
NA	LKAAAAAAKLAAKAAKAALKAAAAAAKL	3.2	8.1
ATCC 19606	WGRRWRIRIPRLPRPWPPRPKWPRSATINTDQ	2.0	8.1
NA	KRGFGKKLRKRLKKFRNSIKKRLKNFNVVIPIPLPG	1.9	8.1
NA	AMVGT	17.1	8.2
NA	GMASKAGSVLGKVAKVALKAAL	4.0	8.3
ATCC 19606	FLSGIVGMLGKLF	6.0	8.3
ATCC 19606	FLKGIVGMLGKLL	6.0	8.3
ATCC 19606	ALWRRLLRRLLRSARRLG	3.8	8.5
NA	FFPVIGRILNGIL	6.0	8.8
ATCC 19606	FLKGIVGMLGKLW	6.0	8.8
ATCC 15308	WPKRLTKAHWFEIQHIQPSPLQCNRAMSGINNYTQHCKHQ NTFLHDSFQNVAAVCDLLSIVCKNRRHNCHQSSKPVNMTD CRLTSGKYPQCRYSAAAQYKFFIVACDPPQKSDPPYKLVPVH LDSIL	0.6	9.1
NA	GILKTIKSIASKVANTVQKLKRKAKNAV	3.1	9.3
NA	FALGAVTKRLPSLFCLITRKC	4.0	9.4
NA	KRRGSVTTRYQFLMIHLLRPKKLFA	3.1	9.4
NA	RVRRFWPLVPVAINTVAAGINLYKAIRRK	2.8	9.5
NA	IKLSPKTKDNLKKVLKGAIKGAIAVAKMV	3.1	9.5
NA	IKLSPKTKKNLKKVLKGAIKGAIAVAKMV	3.1	9.5
NA	IKLSPETKKNLKKVLKGAIKGAIAVAKMV	3.1	9.5
NA	GILKTIKSIASKVANTVQKLKRKAKNAVA	3.1	9.5
NA	IKLSKKTKDNLKKVLKGAIKGAIAVAKMV	3.1	9.6
NA	IKLSKETKKNLKKVLKGAIKGAIAVAKMV	3.1	9.6
ATCC 19606	FVQWFSKFLGKIL	6.0	9.7

ATCC 19606	LLQWLSKLLGRWL	6.0	9.8
ATCC 19606	FVQWFSKFLGRIL	6.0	9.8
ATCC 19606	LIRGLFKSFWQVF	6.0	9.8
CCARM 12036	FAHHIFRGIVHVGKTIHRLVTG	4.0	10.0
CCARM 12036	FFHHIARGIVHVGKTIHRLVTG	4.0	10.0
NA	KIKKGFKKIFKRLPPIGVGVSIPLAGKR	3.3	10.0
NA	GLFKKLRRKIKKGFKKIFKRL	3.8	10.0
ATCC 19606	FVQWFSRFLGRIL	6.0	10.0
ATCC 19606	FVRWFSKFLGRIL	6.0	10.0
ATCC 19606	FVRWFSRFLGRIL	6.0	10.2
CCARM 12036	FFHHIKRGIVHVGKTIHRLVTG	4.0	10.2
CCARM 12036	FFHHIKRGIKHVGKTIHRLVTG	4.0	10.3
ATCC 19606	FFGRLKSVWSAVKHGWKAAKSR	4.2	10.8
NM8	GFLGSLLKTGLKVGSNLL	6.0	10.9
NA	GFWGKLWEGVKNAI	7.0	11.2
NM8	GVIKSVLKGVAKTVALGML	6.0	11.3
NM8	GLKEIFKAGLGSLVKGIAAHVAS	5.0	11.3
NA	GLGSLLGKAFKIGLKTVGKMMGGAPREQ	4.0	11.4
NM75	GLKKIFKAGLGSLVKGIKAHVAS	5.0	11.6
NA	GIGAVLKVLTTGLPALISWIKRKRQQ	4.2	12.1
NA	KWKLFKKIPKFLHLAKKF	5.4	12.5
ATCC 19606	FLSMIPHIVSGVAALAKHL	6.3	12.5
ATCC 15308	ISLNPPRSTIAMRAINNYRWRSKNQNTFLR	3.5	12.7
ATCC 19606	FLSLIPHIVSGVASIAKHF	6.3	12.7
ATCC 19606	FLSLIPHIVSGVASLAKHF	6.3	12.7
NM8	ILGKLLSTAAKLLSNL	8.0	13.2
NA	IASKVANTVQKLKRKAKNAVA	6.3	14.0
NA	ILSAIWSGIKSLF	10.0	14.3
ATCC 17978	NPEKALEKLIAIQKAIKGMLNGWFTGVGFRRKR	4.0	15.1
ATCC 19606	FFHHIFRPIVHVGKTIHRLVTG	5.9	15.4
NA	IKSIASKVANTVQKLKRKAKNAV	6.3	15.6
ATCC 15308	GLFDIWKWWRWRR	8.2	15.6
NM35	IKIPSFFRNILKKVGKEAVSLIAGALKQS	5.0	15.8
NA	KWCFRVCYRGICYRKCR	7.1	16.0
NA	VQLRIRVCVIRK	10.8	16.0
Q12	TMSLRFWRWKVR	9.6	16.0
ATCC 19606	KILGVSKKIMRRISKDILTGKK	6.4	16.0
Q12	QVRWWGRYWRRKWATCR	6.7	16.0
AB1	QKKIRVRLSA	13.4	16.0
NA	KRIVQRIKDFLRNLVPRTES	6.5	16.0
DSM 30008	VDKPPYLPRPRPPRRIYNR	6.7	16.0
NA	VQWRIRIAVIRA	10.8	16.0
NA	KRFKKFFKKLKNSVKKRAKKFFKKPRVIGVSIPF	3.9	16.0
ATCC 19606	GKKYRRFRWKFRKGRFWFWG	5.7	16.0
ATCC 19606	GRRYKKFRWKFKGRWFWFG	6.1	16.0
ATCC 19606	GKKYRRFRWKFKGKWFWFG	6.1	16.0
ATCC 19606	LLWKALRLWWKVL	9.3	16.0
ATCC 19606	GKKYRRFWKFKGKWFWFWG	6.1	16.0
ATCC 19606	WFWKLLWKALRLWWKVL	6.7	16.0

ATCC 19606	GCRALCYKQRCVTYCRGA	8.0	16.4
ATCC 19606	LLKLLKKLLKKLLKL	10.0	18.1
ATCC 19606	YSWPRMPRIPRLPRYPRYPRYPRWPRWPRQPTIYA	4.0	18.5
ATCC 19606	LLQWLSKLLGRLL	12.0	18.6
NA	IKLSPETKDNLKKVLKGAIKGAIAVAKMV	6.3	19.2
NA	IKLSKETKDNLKKVLKGAIKGAIAVAKMV	6.3	19.3
ATCC 19606	FLPWFSKFLGRIL	12.0	19.5
ATCC 19606	KKEK	37.0	19.7
ATCC 19606	LLKKLLKLLLKLLKK	11.0	19.9
CICC 22934	SKVWRHWRRFWHRAHRKK	7.8	20.0
NA	LLKKLLKKC	18.4	20.0
ATCC 19606	FVPWFSKFLGRIL	12.5	20.1
NA	IWSAIWSGIKGLL	14.0	20.2
ATCC 19606	KK	75.0	20.6
CCARM 12036	GLNALKKVFQPIHEAIKLINNHVQ	8.0	21.8
CCARM 12036	GLNALKKVFQPIHKAIKKINNHVQ	8.0	21.9
ATCC 19606	LRWTPTPSYPRYPTRSRGSRWSR	8.0	22.9
NM8	GFSSIFRGVAKFASKGLGKDLAKLGVDLVACKISKQC	6.0	23.1
ATCC 15308	RPPQFTRAQWFAIQHISLN	10.0	23.1
NA	GIWSSIKNLASKAWNSDIGQSLRNKAAGAINKFVADKIGVTP SQAAS	5.0	24.4
NA	RRSKARGGSRGSKMGRKDSKGGSRGRPGSGSRPGGGSSIAGA SRGDRGGTRNA	4.7	24.4
ATCC 19606	WLRRIKAWLRR	16.0	24.9
NA	FLPAALAGIGGILGKLF	15.8	26.2
ATCC 19606	KLKLLLKLLKLLLKLLLK	15.0	27.1
ATCC 19606	KGGGKWGGKGGK	25.0	27.9
ATCC 19606	KKLLKKLKKLLK	19.0	28.1
NA	GIWDTIKSMGKVFAGAILQNL	12.5	28.3
NA	GIWKTIKSMGKVFAGAILQNL	12.5	28.4
ATCC 19606	EKALEKLIAIQKAIKGMLNGWFTGVGFRRKR	8.0	28.5
NM35	GIFPIFAKLLGKVIKVASSLISKGRTE	10.0	28.7
ATCC 17978	KRWWKWWRR	20.0	29.8
ATCC 15308	FLGGLIKWWPWRR	18.2	31.3
ATCC 15308	FLGGLIKWKWPWWPWRR	13.5	31.3
ATCC 15308	GLFDIWKKLRWRR	17.6	31.3
ATCC 15308	GLFDIWKKWRWRR	16.9	31.3
ATCC 15308	GLFDIWAWWRWRR	16.9	31.3
ATCC 19606	ALWHHLLHHLLHSAHHLG	15.0	31.9
NA	CLRKLRKRLLC	22.8	32.0
SR 201346	FLGGLIKIVPAMICAVTKKCHHHHHH	10.9	32.0
NA	VQLRIRVCVIRR	21.2	32.0
Q12	AWRWKAFRNCWRVRSSSL	13.9	32.0
NA	VQLRIRVAVIRA	23.0	32.0
ATCC 19606	KFHEKHHSHRGY	20.5	32.0
NA	LRKLRKRLLLRKLRKRLL	13.5	32.0
AB1	VAKGLIKGVKAKGELPAKGVFKGLKESIGKRAVLKG	8.7	32.0
NA	LLKKALRLWKKVL	19.9	32.0
Q12	SIKILKIYFIQGKRHWSF	14.1	32.0

NA	GFWSSALEGLKKFAKGGLEALTNPK	12.5	33.1
NA	FLPLIGRVLSGIL	24.0	33.5
ATCC 19606	KAK	100.0	34.5
ATCC 19606	FLKGIKGMLGKLL	25.0	35.4
ATCC 19606	FLKGIVGKLGKLF	25.0	35.5
NA	GFLGPLLKLGLKGVAKVLPHLIPSRQQ	12.5	36.0
ATCC 19606	FLKGIKGMLGKLF	25.0	36.3
ATCC 19606	GIGK	100.0	37.3
NA	GWANTLKNVAGGLCKITGAA	19.3	37.5
ATCC 19606	FLFSLIPSAIGGLISAFK	20.0	37.6
ATCC 19606	KLK	100.0	38.8
CCARM 12036	GLNALKKVFQPIHEAIKKINNHVQ	16.0	43.8
ATCC 19606	DDALKKLLKKLLKKL	25.0	44.2
NM8	GLKKIFKAGLGSLKKGIAAHVAS	20.0	45.9
NM8	GFSSIFRGVAKFASKGLGKDLAKLGVDLVASKISKQS	12.5	47.6
ATCC 19606	GIGKFLHSAKKFGKAFVGEIMNS	20.0	49.3
NM8	GMATKAGTALGKVAKAVIGAAL	25.0	50.0
ATCC 15308	KESRAKKFQRQHMDSDSSPSSSSTYSNQMMRRRNMTQGRSK PVNTFVH	9.0	50.7
NM8	ILGKLLSTAAGLLKNL	32.0	52.0
ATCC 15308	KPKDMTSSQWFKTQHVQPSPQASNSAMSIINKYTERSKDLNT FLH	10.0	52.1
ATCC 15308	KPPQFTWAQWFETQHINMTSQQSTNAMQVINNYQRRSKNQ NTFLL	10.0	54.6
ATCC 15308	QDNSRYTHFLTQHYDAKPQGRDDRYSESIMRRRGLTSPSKDI NTFIH	10.0	56.4
ATCC 19606	WW	145.5	56.8
NA	GIWDTIKSMGKVFAGLILQNL	25.0	57.6
ATCC 19606	KGIVGMLGKLF	50.0	58.1
NA	FWGKLWEGVKNAI	38.0	58.8
NA	LGAWLAGKVAGTVATYAWNRYV	25.0	59.2
NA	GLASTIGSLLGKFAKGGAQAFLQPK	25.0	61.5
ATCC 15308	GLFDKWAWWRWRR	33.5	62.5
ATCC 15308	FLGGLIKWPWWPWRR	31.3	62.5
NA	SKVWRHWRRFWHRAHRLH	25.0	63.8
ATCC 19606	GCRRWKKFRWRYRGKFWFWCG	22.0	64.0
ATCC 19606	GCRRFKKFKKWRYRGRFWFWCFG	20.3	64.0
Q12	KFVRLKIYCRDKNKGRGISF	26.3	64.0
ATCC 19606	GIGKFLHSAGKFGKAFVGEIMKS	26.6	64.0
ATCC 19606	GCKKYRRFRWKFKGKFWFWGG	23.1	64.0
ATCC 19606	GCKKYRRFRWKFKGKFWFW	24.1	64.0
Q12	VLHTGYRKFLHRSKRFFHLR	24.6	64.0
NA	KLLK	127.8	64.0
DSM 3008	TWLKKRRWKKVKPP	34.6	64.0
ATCC 19606	KKKLKKLKKKLK	50.0	75.6
ATCC 19606	FVQWFSKFLLRIL	48.0	81.5
ATCC 19606	FVPWFSKFLPRIL	50.0	82.5
DSM 30007	RWRWRW	85.0	88.8
ATCC 19606	DDALKHLLKHLLKHL	50.0	89.7

ATCC 19606	KKKLLLLLLLLLKKK	50.0	90.3
ATCC 19606	LLLLLKKKKKKLLLL	50.0	90.3
NA	GLVGTLLGHIGKAILG	62.5	94.9
NA	GLVGTLLGHIGKAILS	62.5	96.8
NA	KVANTVQKLKRKAKNAVA	50.0	98.4
NM8	ILGKLLSTAAGLLSNL	64.0	101.4
ATCC 19606	FSFLSRIF	100.0	101.6
NM8	ILGKLLKTAAGLLSNL	64.0	104.0
ATCC 19606	FFFLSRIF	100.0	107.6
NA	SAVGRHGRRFGLRKHRKH	50.0	107.8
NA	IASKVANTVQKLKRKAKNAV	50.0	108.4
NA	LKFLKFG	128.0	109.1
ATCC 19606	FFFLRRIF	100.0	114.5
NA	LKWLKWG	128.0	119.1
ATCC 19606	KAAAKWAAKAAK	100.0	121.4
ATCC 15308	FLGGLIKPWWPWRR	69.0	125.0
NA	LRWLRWG	128.0	126.2
NA	LRDLVCYCRTRGCKRRERMNGTCRKGHLMYTLCCR	30.1	128.0
NA	GIGDPVTCLKSGAICHPVFCPRRYKQIGTCGLPGTKCCKKP	29.5	128.0
ATCC 19606	KKIMRTFLRR	94.9	128.0
ATCC 19606	SMATPHVAGAAALILSKHPTWTNAQVRDRLESTATYLGNSF YYGK	26.1	128.0
ICU 63169	PRPRPRP	146.3	128.0
ICU 63169	PRPGPRP	165.0	128.0
ATCC 19606	KILRGVSKRILTGKK	75.4	128.0
Q13	GTAWRWHYRARS	82.8	128.0
ICU 63169	PRPLPRP	153.8	128.0
NA	AKRHHGYKRKFH	81.8	128.0
Q13	RSITRPVLVRRRWRVRPVF	52.2	128.0
ATCC 19606	KWKIFKKIEKVGRNIRNGIIKAGPAVAVLGEAKAL	33.5	128.0
ICU 63169	PRPWPRP	141.4	128.0
ATCC 19606	AGYLLPKINLKPLAKLPKKIL	54.9	128.0
NA	KRFKKFFKKVKKSV	71.2	128.0
ATCC 19606	KVVVKWVVKVVK	100.0	141.1
ATCC 19606	WPRFPKPRKPTYPGPTYPGPTWPRPTWRRSATIDTEH	32.0	141.8
ATCC 19606	KLLLKWLLKLLK	100.0	150.9
NM8	GFSSIFRGVAKFASKGLGKDLAKLGVDLVA	50.0	152.6
ATCC 19606	DDALHHLLHHLLHHL	100.0	182.1
ATCC 19606	DDALRHLLRHLLRHL	100.0	187.8
ATCC 19606	DDALRRLLRRLLRRL	100.0	193.5
NA	KKCGFFCKLKNKLKSTGSRSNIAAGTHGGTFRV	56.4	200.0
NA	KKCKFFCKVKKKIKSIGFQIPIVSIPFK	60.9	200.0
NA	TRWLWLLRGGLKAAGWGIRAHLNRNQ	65.7	200.0
NA	KFFKRLLKSVRRAVKKFRKKPRLIGLSTLL	55.1	200.0
NM8	ILGKLLSTAAGLLSKL	128.0	204.5
CICC 22934	SKVGRHGRRFGHRAHRKL	100.0	215.6
NM8	ILGAILPLVSGLLSNKL	128.0	220.3
CICC 22934	SAVGRHLRRFGLRKHRKH	100.0	221.2
CICC 22934	SKVGRHLRRFGHRAHRKL	100.0	221.2

CICC 22934	SAVGRHLRRFLLRKHRKH	100.0	226.8
CICC 22934	SKVGRHLRRFLHRAHRKL	100.0	226.8
CICC 22934	SAVLRHLRRFLLRKHRKH	100.0	232.4
CICC 22934	SKVLRHLRRFLHRAHRKL	100.0	232.4
ATCC 19606	NPEKALEPLIAIQIAIKGMLNGWFTGVGFRRKR	64.0	238.4
ATCC 15308	FLGGLIKRPPAMICAVRKKC	113.5	250.0
ATCC 15308	FLGGLIKRVPAMICAVRKKC	113.4	250.0
ATCC 15308	FLGGLIKPVPAMICAVRKKC	116.6	250.0
ATCC 15308	FLGGLIKIVPAMICAVRKKC	115.7	250.0
NA	PPPVIKFNRPFLMWIVERDTRSILFMGKIVNPKAP	60.8	250.0
ATCC 15308	RLPWRWPRRPWRR	130.3	250.0
NA	KTRNWFSEHFKKVKEKLKDTFA	90.3	250.0
NA	FSTKTRNWFSEHFKKVKEKLKDTFA	80.6	250.0
ATCC 15308	RRPWRWPRWPWRR	125.5	250.0
ATCC 15308	RRPWRWPWWPWRR	123.7	250.0
ATCC 15308	LLPWKWPWWKWRR	129.0	250.0
ATCC 19606	FASGIAGMAGKLF	200.0	253.9
ATCC 19606	DHYNCVSSGGQCLYSACPIFTKIQGTCYRGKAKCCK	65.1	256.0
ATCC 19606	KISKKIMRTFLRR	152.6	256.0
ATCC 19606	AGRGKQGGKVRAKAKTRSSRAGLQFPVGRVHRLLRKGNY	60.1	256.0
ATCC 19606	ISKRILTGKK	223.9	256.0
ATCC 19606	FAKGIAGMAGKLF	200.0	262.1
NM124	KFASKGLGKDLAKLGVDLVACKISKQC	100.0	282.1
ATCC 19606	KLAKLAKKLAKLAK	196.9	300.0
ATCC 19606	AVAGEKLWLLPHLLKMLLTPTP	163.8	400.0
ATCC 19606	FLSLIPHIVSGVASLAIHF	200.0	404.3
ATCC 19606	WKRRIKIWKKIR	256.0	438.1
NM8	GLGKDLAKLGVDLVACKISKQC	200.0	452.0
NM8	KGLGKDLAKLGVDLVACKISKQC	200.0	477.6
NM35	RGVAKFASKGLGKDLAKLGVDLVACKISKQC	200.0	641.0
